# Burden of non-communicable diseases attributable to dietary risks in Brazil, 1990-2019: an analysis of the Global Burden of Disease Study 2019

**DOI:** 10.1590/0037-8682-0282-2021

**Published:** 2022-01-28

**Authors:** Ísis Eloah Machado, Magda do Carmo Parajára, Larissa Fernanda Fonseca Guedes, Adriana Lúcia Meireles, Mariana Carvalho de Menezes, Mariana Santos Felisbino-Mendes, Eliseu Verly-Junior, Deborah Carvalho Malta

**Affiliations:** 1 Universidade Federal de Ouro Preto, Departamento de Medicina de Família, Saúde Mental e Coletiva, Ouro Preto, MG, Brasil.; 2 Universidade Federal de Ouro Preto, Programa de Pós-Graduação em Saúde e Nutrição, Ouro Preto, MG, Brasil.; 3 Universidade Federal de Ouro Preto, Departamento de Nutrição Clínica e Social, Ouro Preto, MG, Brasil.; 4 Universidade Federal de Minas Gerais, Programa de Pós-Graduação em Enfermagem, Belo Horizonte, MG, Brasil.; 5 Universidade do Estado do Rio de Janeiro, Instituto de Medicina Social, Departamento de Epidemiologia, Rio de Janeiro, RJ, Brasil.

**Keywords:** Nutritional epidemiology, Diet, food, and nutrition, Global Burden of Disease, Mortality, Disability-adjusted life years, Risk factors

## Abstract

**INTRODUCTION:**

An unhealthy diet is a modifiable risk factor for non-communicable diseases (NCDs), one of the most important public health problems in Brazil. This study aimed to analyze the burden of NCDs attributable to dietary risks in Brazil between 1990-2019.

**METHODS:**

Secondary data from the Global Burden of Disease Study were used to estimate the burden attributable to fifteen dietary risks in Brazil. The main sources of data for Brazil were national surveys and international databases. A comparative risk assessment was used to obtain the population attributable fraction. We described the intake of each dietary risk and the distribution of number and rates of deaths and Disability-adjusted life years (DALYs) attributable to diet by sex, age, state, and year from 1990-2019.

**RESULTS:**

Cardiovascular diseases, diabetes mellitus, and neoplasms were the main NCDs attributable to an unhealthy diet. Age-standardized mortality and DALYs rates attributable to unhealthy diet decreased between 1990-2019 (-51.5% and -48.8, respectively). Diet high in red meat and **s**odium, and low in whole grains were the three main risk factors contributing to the burden of NCDs both in 1990 and 2019. The burden of NCDs was higher among males in the middle-aged population (around 50 years), as well as in the states of Maranhão, Rio de Janeiro, and Alagoas.

**CONCLUSIONS:**

The present study found a suboptimum diet among the Brazilian population. The major contributors to this burden were diet high in red meat and sodium and low in whole grains. This study supports priorities in public policies on food and nutrition to reduce the burden of NCDs.

## INTRODUCTION

Non-communicable diseases (NCDs) configure a huge public health problem^1^ ^,^ ^2^, with diabetes mellitus, cardiovascular diseases (CVDs), neoplasms, and chronic respiratory diseases being responsible for approximately 74% deaths worldwide^2^ ^,^ ^3^. Data from the Global Burden of Disease (GBD) study for Brazil have shown that the proportional mortality due to NCDs in 2017 corresponded to 75.9%, while the proportion of premature deaths due to this disease group was 28%^4^.

As NCDs result in premature deaths and provide a high degree of limitation and inability to perform daily activities, this burden leads to a loss in the quality of life for individuals and families, causes an overload on health systems, and has a negative impact on the country's economy. It is important to emphasize that, although NCDs affect all socioeconomic strata, the disease burden falls more intensely on low- and middle-income countries, especially among those individuals belonging to vulnerable groups, with low education and lower income levels^1^ ^,^ ^5^ ^,^ ^6^.

Among the main risk behaviors related to NCDs morbidity and mortality, four modifiable risk factors stand out: inadequate diet, insufficient physical activity, tobacco use, and harmful consumption of alcoholic beverages^1^. Regarding the dietary pattern of the Brazilian population, in recent decades, an increase has been observed in ultra-processed food consumption and a decrease in the consumption of fresh foods, resulting in inadequate and, in general, highly energy-dense and nutrient-poor diets^7^. Low- and middle-income countries, such as Brazil, where there are vulnerabilities in food access, have been experiencing a coexistence of malnutrition with undernutrition, overweight, obesity, and NCDs^8^.

The monitoring of NCDs and their risk factors to better understand their trends have been considered a public health priority and an important action for the development and implementation of effective public policies in Brazil^9^ ^,^ ^10^. Considering the present pandemic scenario, it is already well-known that the severity and mortality of COVID-19 has significantly increased in people with NCDs. Moreover, the ability of countries to address and respond to NCDs has been impacted^11^. Therefore, tackling the major risk factors for NCDs, such as diet, is a constant concern.

Thus, this study aims to analyze the burden of NCDs attributable to dietary risks in Brazil, regarding the deaths and Disability-adjusted life years (DALYs) between 1990 and 2019, according to estimates from the GBD 2019 study.

## METHODS

This study used secondary data publicly available in GBD study 2019, developed by the Institute for Health Metrics and Evaluation (IHME). The data were extracted in February 2021 through the GBD Results Tool (http://ghdx.healthdata.org/gbd-results-tool) for Brazil and its 27 states. 

We analyzed the 15 dietary risks proposed by the GBD 2019 according to sufficient and available data to estimate risk factors exposure^12^. Hence, the dietary risks evaluated were diet low in fruits, vegetables, legumes, whole grains, nuts and seeds, milk, fiber, calcium, seafood omega-3 fatty acids, and polyunsaturated fatty acids (PUFA), and diet high in red meat, processed meat, sugar sweetened beverages (SSBs), trans fatty acids (TFA), and sodium^13^.

The GBD 2019 estimated the exposure of the Brazilian population to each risk, based on dietary recall sources obtained from systematic reviews, national surveys conducted by the Brazilian Ministry of Health (MoH), and the Brazilian Institute of Geography and Statistics (IBGE), such as the Surveillance System for Risk and Protective Factors for Chronic Diseases by Telephone Survey (Vigitel) and the Consumer Expenditure Survey (POF), and available data from the Food and Agriculture Organization of the United Nations (FAO) Supply and Utilization Accounts. When local data is not available, GBD may also use sources from other countries^13^.

Since the data sources to estimate nutrient intake may have specific biases, a network meta-regression (MR-BRT) was applied in GBD 2019 to adjust the data obtained by different methods and make them comparable with those from the gold standard methods^13^. The gold standard data source for all dietary risks is the 24-hour dietary record, except for sodium whose gold standard is the 24-hour urinary sodium (Supplementary Material Table 1). The exposure estimates for each dietary risk, corresponded to the average intake in grams per person per day of each nutrient, by age, sex, year, and location were modeled using a spatiotemporal Gaussian process regression (ST-GPR) framework^12^ ^,^ ^13^.

### Optimal Level of Dietary Risk Factors Intake

In GBD 2019, the theoretical minimum risk exposure level (TMREL) was set to zero for all harmful dietary risks on the rise, except for sodium which was defined in 3 g/day (1-5 g/day)^13^. For dietary factors in which exposure reduces an outcome (such as fruit intake), the 85th percentile of exposure found in the cohorts or trials were used in the meta-regression of each risk-outcome pair. Therefore, the TMREL was calculated using the average of these numbers and weighing them by the relative global magnitude of each outcome^13^. The TMREL applied for each dietary risk can also be found in the Supplementary Material Table 1.

### Burden of Disease Attributable to Dietary Risks

Risk-outcome pairs were selected in the literature based on most recent systematic reviews and meta-analysis with the aim of identifying epidemiological evidence to support the causal relationship between the 15 dietary risks and their respective health outcomes (the risk-outcome pairs are shown in the Supplementary Material Table 2). After that, relative risks for each risk-outcome pair by age, sex, and location were obtained by meta-analysis derived from cohort studies or trials included in the GBD 2019 publications. The size of the effect of the associations and uncertainties for each cause were also estimated by meta-regression. The population attributable fraction (PAF) was calculated using the information from the counterfactual scenario of TMREL. The proportion of each dietary risk on its related outcome was then estimated by multiplying the PAF by the disease burden envelope^12^ ^-^ ^14^. 

### Data Analysis

The burden of disease in this study was measured using deaths and DALYs stratified by sex, age group, and states, in 1990 and 2019. The estimates were represented through absolute numbers, proportions, and age-standardized rates per 100,000 inhabitants. Age-standardized rates in the GBD were calculated using the standard GBD world population. The investigated population consisted of Brazilian adults, aged 25 years or older.

Considering the analytical strategy, we first presented the estimated mean daily intake of each dietary risk in 2019. The number of deaths and DALYs in 2019 were then stratified by each specific outcome. The deaths and DALYs rates from 1990 to 2019, together with the percentage change over the period, were also presented. The proportion of deaths and DALYs due to non-communicable diseases attributable to dietary risks in 2019 were analyzed by sex, age, and for each state.

The 95% uncertainty intervals (95% UI) were estimated from 1,000 calculations of each parameter distribution in each iteration. The mean and its lower and upper limits were calculated based on the 2.5 and 97.5 percentiles^12^. 

## RESULTS

In 2019, the intake of healthy foods and nutrients, such as fruits, milk, whole grains, vegetables, calcium, fiber, seafood omega-3 fatty acids, and PUFA were suboptimum (lower than TMREL) among Brazilian adults aged 25 years or older. Legumes, nuts, and seeds were the only healthy dietary factors with intakes higher than TMREL. The consumption of all the unhealthy dietary factors (processed and red meat, SSBs, TFA, and sodium) exceeded the minimal risk level. 

**FIGURE 1:**
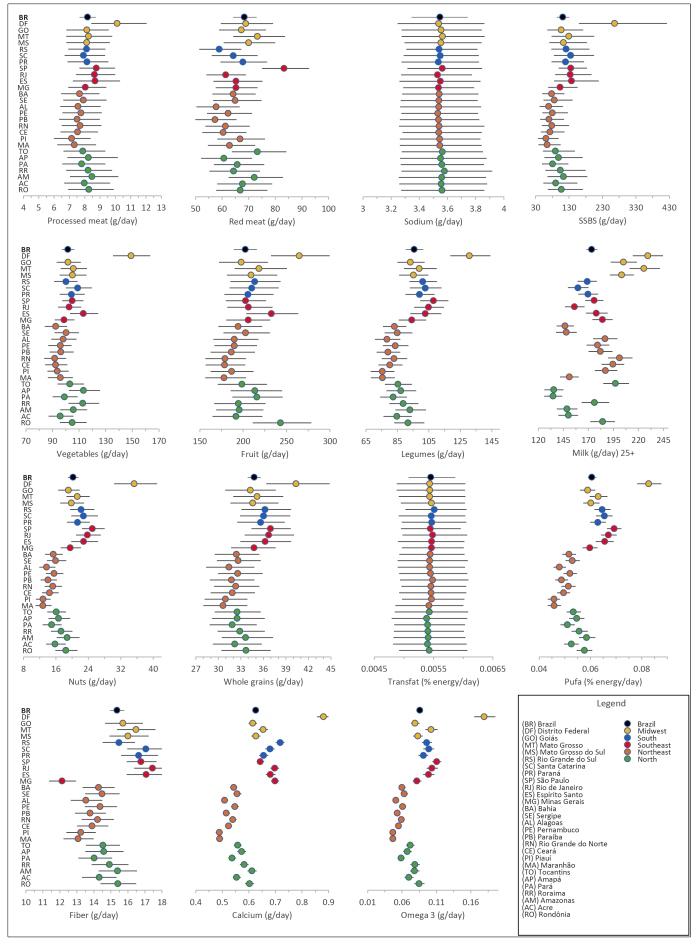
Mean intake of dietary factors among adults aged 25 years or older in Brazil and its states in 2019.

We highlight the higher consumption of healthy nutrients, such as fiber, calcium, omega-3, and PUFA in the Southeastern, Southern, and Midwestern states. Milk consumption was high in the Midwestern states. The lowest average vegetable, nuts and seeds, PUFA, calcium, and omega-3 consumptions were observed in the Northeastern states. The Federal District stands out for the high average consumption of vegetables, fruit, legumes, nuts and seeds, whole grains, PUFA, calcium, and omega-3, as well as for the SSBs. In São Paulo, red meat was the most prominent (Figure 1; Supplementary Material Table 3).

**FIGURE 2:**
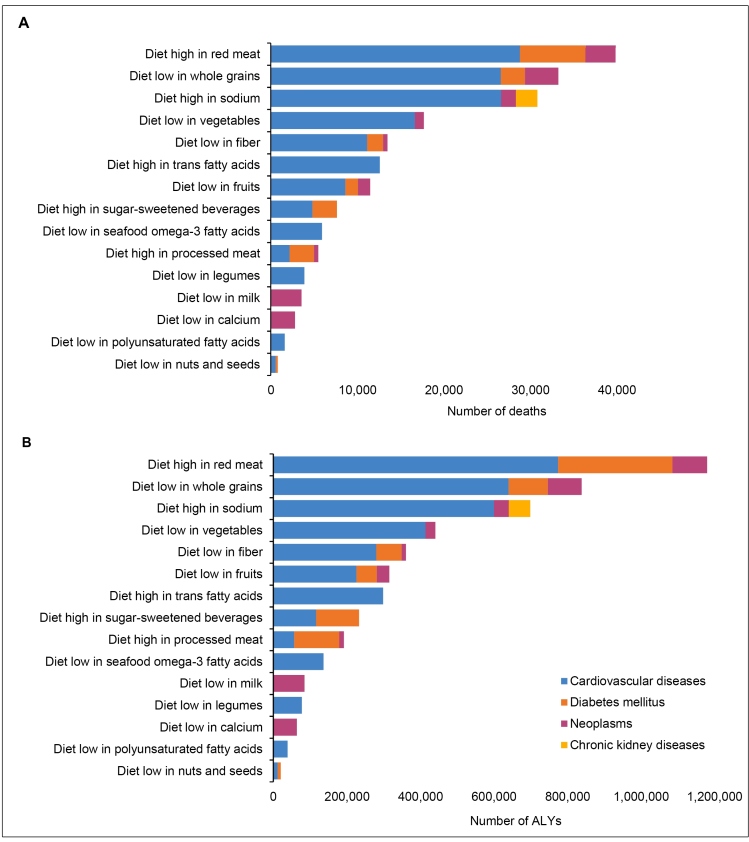
Number of deaths (A) and DALYs (B) due to non-communicable diseases attributable to dietary risks for both sexes and all ages in Brazil, 2019.

**Legend: DALYs:** Disability-adjusted life years.

Diet high in red meat, followed by a diet low in whole grains and high in sodium, were the main causes of NCDs deaths and DALYs for both sexes and all age groups in 1990 and in 2019. The leading cause of deaths and DALYs were CVDs, diabetes mellitus, and neoplasms (Figure 2; Supplementary Material Table 4). 

**FIGURE 3:**
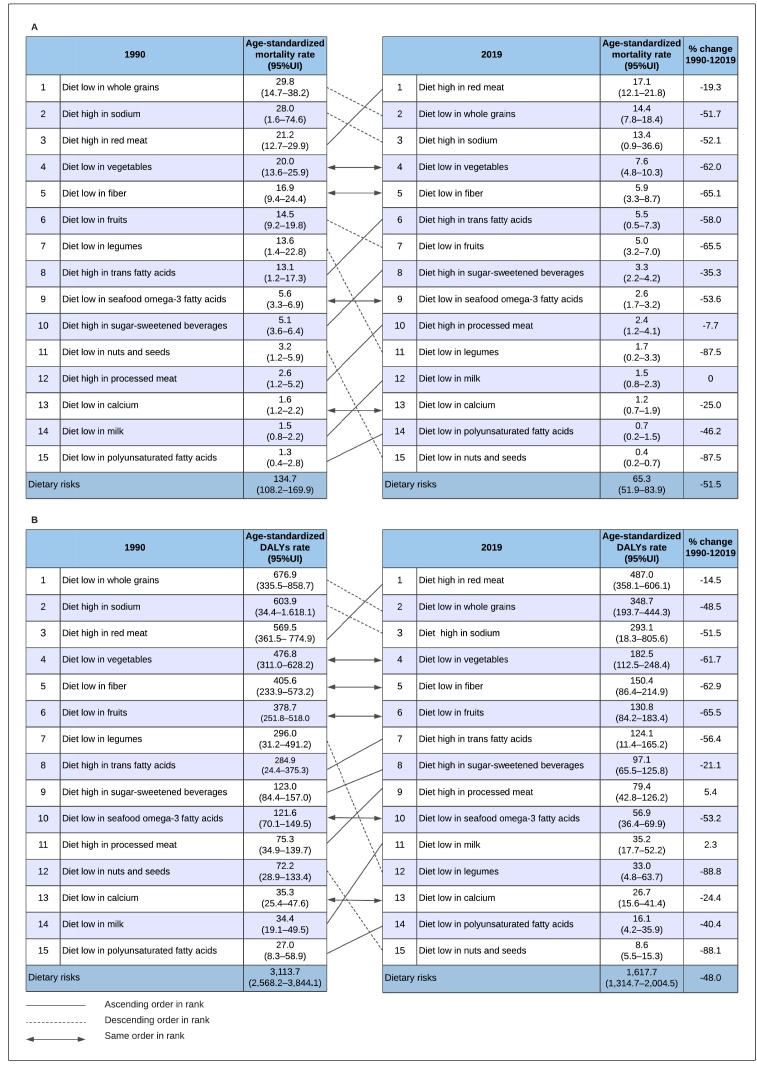
Ranking of the age-standardized mortality rate per 100,000 inhabitants (A) and DALYs rate per 100,000 inhabitants (B) due to non-communicable diseases attributable to total and individual dietary risks for both sexes in Brazil and the percentage change from 1990 to 2019.

**Legend: 95% UI:** 95% Uncertainty interval. **DALYs:** Disability-adjusted life years.

The age-standardized mortality and DALYs rates for the total and the 15 individual leading dietary risks are ranked in Figure 3. There was a total of 134.7 (95% UI: 108.2-169.9) and 65.3 (95% UI: 51.9-83.9) deaths per 100,000 inhabitants due to NCDs attributable to dietary risks in 1990 and 2019, respectively, with a -51.5% reduction between 1990 and 2019. The exception was for low milk intake, which remained stable during the period. The highest age-standardized mortality declines were observed for diets low in legumes (-87.5%), nuts and seeds (-87.5%), and fruits (-65.5%). Low intake of whole grains was the leading risk factor in 1990 (29.8; 95% UI: 14.7-38.2 deaths per 100,000 inhabitants), followed by a high intake of sodium and red meat. In 2019, the highest rates were attributed to a high intake of red meat (17.1; 95% UI: 12.1-21.8 deaths per 100,000 inhabitants), a low intake of whole grains, and a high intake of sodium (Figure 3A). 

When compared to age-standardized mortality rates, trends in age-standardized DALYs rates were similar. The age-standardized DALYs rates of NCDs attributable to dietary risks was 3,113.7 (95% UI: 2,568.2-3,844.1) per 100,000 inhabitants in 1990 and 1,617.7 (95% UI: 1,314.7-2,004.5) per 100,000 inhabitants in 2019, corresponding to a -48.8% reduction over the period. The highest reductions were observed for a diet low in legumes (-88.8%), nuts and seeds (-88.1%), and fruits (-65.5%). On the other hand, positive trends were observed for a diet high in processed meat (5.4%) and low in milk (2.3%) (Figure 3B).

Although mortality and DALYs rates due to NCDs attributable to a diet high in TFA, SSBs, and processed meat have declined from 1990 to 2019, they did rise some positions in the ranking over time (Figure 3A; Figure 3B). 

**FIGURE 4:**
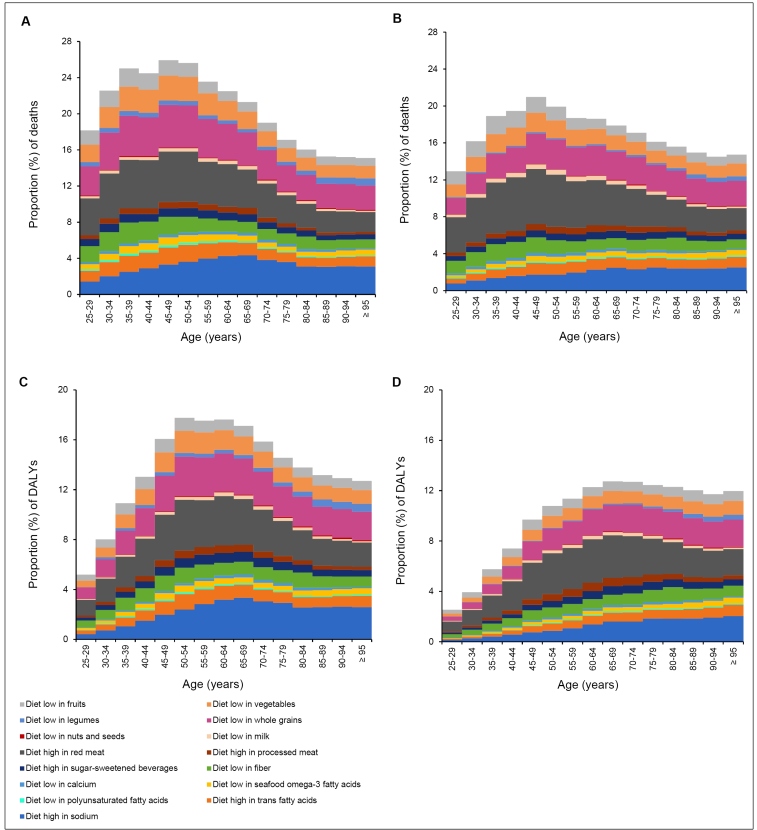
Proportion of deaths among male (A) and female (B) individuals and proportion of DALYs among male (C) and female (D) individuals due to non-communicable diseases attributable to dietary risks by sex and age in Brazil, 2019.

**Legend: DALYs:** Disability-adjusted life years.

Figure 4 shows the proportion of deaths and DALYs due to NCDs attributable to individual dietary risks by sex and age in 2019 (Supplementary Material Table 5). Compared to women, the proportion of deaths caused by dietary risks were higher among men. The highest proportion of deaths for both male and female individuals were between 45 and 49 years of age. The highest proportion of deaths by dietary risks in both sexes and almost all age groups were due to a high intake of red meat, followed by a low intake of whole grains and a high intake of sodium (Figure 4A; Figure 4B). When evaluating the proportion of DALYs, we also found that it was higher among men when compared to women. Additionally, it was observed that males, aged between 50 and 54 years, and females, aged between 65 and 69 years, presented the highest proportion of DALYs attributable to dietary risks. As observed for the proportion of deaths, diets high in red meat, low in whole grains, and high in sodium were the leading dietary risks contributing to the proportion of DALYs in both sexes and in almost all age categories (Figure 4C; Figure 4D).

**FIGURE 5:**
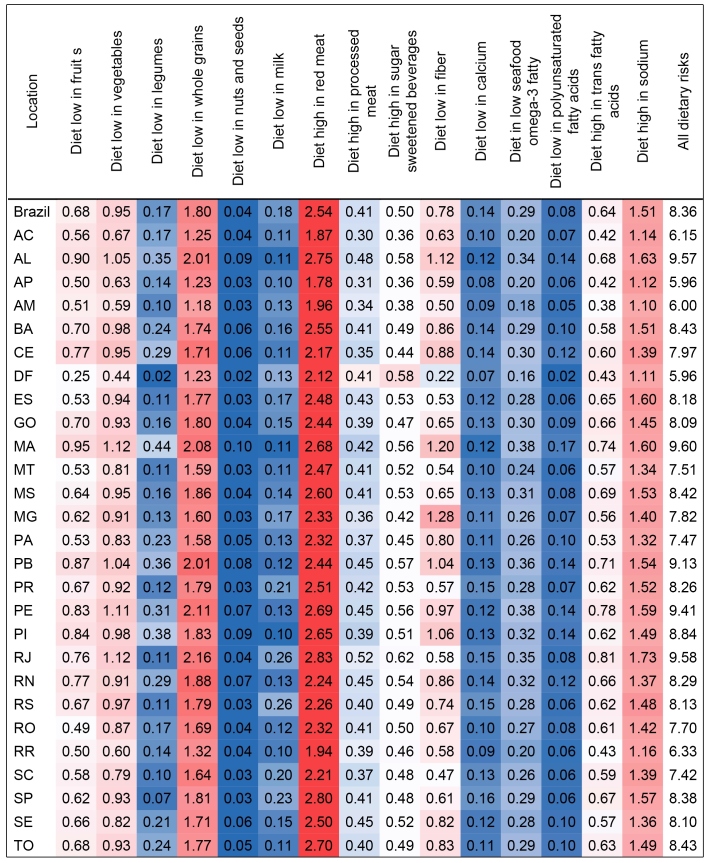
Proportion of disability-adjusted life years (DALYs) due non-communicable diseases attributable to dietary risks for both sexes and all ages in Brazil its states, 2019. **Legend: AC:** Acre; **AL:** Alagoas; **AP:** Amapá; **AM:** Amazonas; **BA:** Bahia; **CE:** Ceará; **DF:** Distrito Federal; **ES:** Espírito Santo; **GO:** Goiás; **MA:** Maranhão; **MT:** Mato Grosso; **MS:** Mato Grosso do Sul; **MG:** Minas Gerais; **PA:** Pará; **PB:** Paraíba; **PR:** Paraná; **PE:** Pernambuco; **PI:** Piauí; **RJ:** Rio de Janeiro; **RN:** Rio Grande do Norte; **RS:** Rio Grande do Sul; **RO:** Rondônia; **RR:** Roraima; **SC:** Santa Catarina; **SP:** São Paulo; **SE:** Sergipe; **TO:** Tocantins. **Note**: The dark red indicates the highest proportions of total DALYs in each location. The dark blue indicates the lowest proportion of total DALYs in each location.

According to Figure 5, the proportion of the total burden of DALYs due to NCDs, for both sexes and all ages in Brazil was 8.36%. Maranhão, Rio de Janeiro, and Alagoas presented the highest proportions of DALYs attributable to dietary risks in 2019 (9.60%, 9.58%, and 9.57%, respectively). The 95% UI of the proportion of DALYs and the proportion of deaths in Brazil and its states in 2019 can be found in the Supplementary Material Tables 6 and 7.

## DISCUSSION

The present study evidenced that age-standardized mortality and DALYs rates due to NCDs attributable to diet in Brazil have decreased from 1990 to 2019. However, Brazilian diet still requires improvements. Among the dietary risks, a diet high in red meat and sodium but low in whole grains were the main contributors to the burden of NCDs. The proportion of diet-related deaths and DALYs was higher in males, in the middle-aged population, and in the states of Maranhão, Rio de Janeiro, and Alagoas. 

Although healthy patterns of diets were observed in several regions, only legumes, nuts, and seeds achieved the optimum intake, according to GBD 2019. One previous study showed a traditional dietary pattern among Brazilian adults, with a high intake of rice and beans, and an increase in the consumption of whole grains, fruits, and vegetables with age; however, young adults reported a high-calorie intake and a poor-nutrient profile in their diets^15^. This means a coexistence of healthy and unhealthy habits in the Brazilian population. Comparing GBD 2019 estimates with the National Dietary Survey (NDS) 2017/2018, a nationwide survey in Brazil that monitors food consumption based on a POF subsample, we found that, although the high intake of beans supports the GBD estimates, the nuts and seeds consumption estimates are quite discrepant^16^. According to the NDS, nuts and seed consumption are not adequate among Brazilians^16^, and these differences need further investigation and adjustments. Additionally, the differences among states could be explained by cultural and sociodemographic determinants^17^.

Our findings also emphasize that approximately 10% of the NCDs could be reduced in Brazil by implementing a healthier diet. It is well-known that diet is a determining factor of human health and well-being, while unhealthy diets are one of the causes associated with the burden of NCDs^18^. However, over the years, changes in the eating patterns have contributed to the occurrence of diet-related NCDs, such as CVDs, diabetes mellitus, and neoplasms^2^ ^,^ ^4^. 

The most important changes in the individuals’ eating patterns have become more evident since the second half of the 20th century, with an increase in ultra-processed foods (UPF) over the traditional eating pattern^19^ ^,^ ^20^. UPF are nutritionally unbalanced and tend to be consumed in large quantities; therefore, their production until consumption are harmful to the heath, society, environment, and culture^21^. Our study showed that diets high in UPF (such as SSBs and processed meat) and nutrients often found in these products (such as TFA) rose in the ranking between 1990 and 2019. Indeed, the caloric share of UPF available in Brazilian households increased between 2002-2003 (12.6%) and 2017-2018 (18.4%)^16^.

*The Eat-Lancet Commission on healthy diets* considers that a healthy and environmentally sustainable diet is based on whole grains, fruits, vegetables, legumes, nuts, and unsaturated oils, including a low to moderate amount of seafood and poultry, but it has little or no amount of red meat, processed meat, added sugar, refined grains, and starchy vegetables^22^. Contrary to these recommendations, our study found diets that are high in red meat, low in whole grains, and high in sodium were the major contributors to the burden of deaths and DALYs due to NCDs, highlighting the need for dietary changes in the Brazilian population. 

In Brazil, the POF showed a reduction in the consumption of beef (21%) and an increase in the consumption of pork (20%) among adults (18-59 years) and the elderly (≥60 years) between the 2007-2008 and 2017-2018 surveys^16^. Nevertheless, the consumption of beef represented the second largest contributor to the total calories among in natura and minimally processed foods^16^. Concerns regarding high meat consumption involve health hazards, as there is strong evidence associating meat consumption with an increased risk of colorectal cancer^23^ ^,^ ^24^. Moreover, red meat production has a higher environmental impact by emitting more greenhouse gases and other pollutants, requiring a large amount of water and causing soil erosion^25^. 

A high intake of sodium is a risk factor for high blood pressure and CVDs^26^. In Brazil, 54.4% of excessive sodium intake was found in the most recent nationwide dietary survey^16^. Moreover, results from the NHS based on urinary sodium excretion has shown that nearly 30% of Brazilians have an intake of above 10g/day, while only 3.4% have a consumption of salt according to the recommended levels^27^. Most of the sodium consumption comes from table salt and salt-based condiments (74.4%); however, between 2002-2003 and 2008-2009 consumption of these ingredients decreased (76.2% to 74.4%), while contribution of processed foods with added salt increased (15.8% to 18.9%) in Brazil^28^. Between 2017-2018 about 20% of the calories in the Brazilian diet came from UPF, which often have a high sodium content^29^, which may in turn explain the burden attributable to this dietary risk found in this study^16^. Approaches aiming to reduce sodium consumption should consider the reduction of salt in preparations and in UPF, as well as in UPF intake^30^ ^,^ ^31^.

A low intake of whole grains is associated with an increased risk of all-cause mortality and NCDs, such as CVD, cancer, and diabetes mellitus^32^ ^-^ ^34^, corroborating our results. In agreement with our finding, the POF 2017-2018 showed that corn, oats, wheat, and other cereals provided only 1.1% of the total calories in the diets^16^. The reasons that may contribute to the lowest consumption of whole grains are their higher cost, greater difficulty to cook and chew, and less attractive colors for some consumers^34^.

Concerning gender, it seems that men are more involved in risk behaviors when compared to women, including inadequate eating patterns^35^ ^,^ ^36^. The Vigitel, a Brazilian telephone survey carried out in the 26 state capitals and the Federal District with individuals aged ≥ 18 years, revealed that 29.8% of the sample consumed five or more unprocessed or minimally processed foods in the day before the interview, with a higher frequency in woman (32.3%) than in men (26.9%)^37^. The same survey also showed that 18.2% of Brazilians consumed five or more UPF, which was higher in men (21.8%) than in women (15.1%)^37^. Compared with men, women have a better quality diet^38^. These differences might be explained by the greater implication of women in food preparation than men and by the concern of women regarding their health, even with the changes resulting from the insertion of women in the labor market^39^.

Our results also showed an increase in deaths and DALYs due to NCDs attributed to dietary risks with increasing age. Exposure to behavioral risk factors, including inadequate nutrition, can start early, consolidate in adulthood, and have negative effects during all life cycles. With population aging and increased life expectancy, reducing the burden of NCDs should be a priority to reduce the demands on the health system, guarantee economic development, and quality of life for individuals^40^ ^-^ ^42^.

The present study found a reduction in the burden of NCDs attributable to dietary risks from 1990 to 2019, reflecting the reduction in the total burden of NCDs in the country^4^. Brazil, along with other countries, has made some commitments over the past few years to face NCDs. The Global Action Plan sets a goal of reducing 25% of premature deaths from NCDs by 2025^43^. Continuing this commitment, the country has also assumed, together with the United Nations (UN), a 30% reduction in premature deaths from NCDs by 2030^44^. Furthermore, we hypothesize that the burden reduction is partly explained by the strategic actions to tackle NCDs, including, for instance, greater access and use of services provided by the Unified Health System (SUS), such as coverage of public programs (for example, the Family Health Strategy), medical consultations, free drug distribution, organization of the emergency care network, diagnosis, and treatment^45^ ^,^ ^46^. 

By contrast, our study’s results elucidate that the Brazilian population’s diet remains inadequate. It should be noted that health promotion goes beyond individual behaviors and does not depend only on the health sector. Much to the contrary, it also depends on intersectoral initiatives that guarantee favorable social and economic conditions for the adoption of a healthy lifestyle. In this direction, regulatory actions are essential, such as controlling advertising aimed at children, taxing UPF, expanding access to healthy foods, inducing policies on food production, distribution and access, educational campaigns, food labeling measures, healthy food supply in schools and workplaces, among others^47^ ^,^ ^48^.

Our study has some limitations. Data on food consumption and NCDs come from multiple sources, and each type of data has specific biases. The lack of data, low response rate, low quality information, and non-inclusion of certain population groups (such as the chronically ill, homeless, and illegal immigrants) can cause errors in the estimates. In addition, the strengths of evidence may vary between food and nutrients, and as a result, there may be an increase in the statistical uncertainty of exposure to dietary risks estimates. Moreover, when estimating the burden of NCDs attributable to dietary risks, the distribution of food factors within each unit of analysis (for example, age and sex) is assumed to be independent, which may result in an underestimation or overestimation of the combined effect of dietary factors^12^.

This study also has key strengths. A consistent and comparable methodology was used to determine the burden of NCDs attributable to dietary risk factors and thus beyond the risk factor prevalence estimation as is generally available. This advance also allows us to estimate the number of deaths and DALYs that could be prevented if these risks were reduced. Our findings reinforce the importance of ongoing actions to reduce premature deaths from NCDs. The use of data from the GBD, a public domain database, will also enable the monitoring of these actions over time. Moreover, these analyses may help to create more targeted financing policies and decisions in the field of food and nutrition with the purpose of promoting better health conditions for the Brazilian population. 
