# Supplementary material for: Burden of non-communicable diseases attributable to dietary risks in Brazil, 1990-2019: an analysis of the Global Burden of Disease Study 2019
Source: Rev Soc Bras Med Trop. 2022 Jan 28;55(Suppl 1):e0282-2021. doi: 10.1590/0037-8682-0282-2021 (PMC9009426; doi:10.1590/0037-8682-0282-2021)
Supplement: Supplementary file 1 [file 1678-9849-rsbmt-55-s01-e0282-2021-supp1.pdf]

# SUPPLEMENTARY MATERIAL

This Supplementary Material provides detailed tables with the description of methodological issues and values for the article “Burden of non-communicable diseases attributable to dietary risks in Brazil, 1990–2019: an analysis of the Global Burden of Disease Study 2019”.

**SUPPLEMENTARY TABLE 1:** Adjustment factors for dietary risk factors applied in the meta-regression (MR-RBT) crosswalk and optimal level of intake (theoretical minimum risk exposure level – TMREL).

| Dietary risk | Sex | Data input | Reference or alternative case definition | Gamma | Beta coefficient, log (95%CI) | Adjustment factor | TMREL per person |
|---|---|---|---|---|---|---|---|
| Calcium | --- | DR | Ref | 0.24 | ---- | --- | Intake of calcium between 1.06 and 1.1 grams per day |
| Calcium | Fem | FAO | Alt | | 0.04 (0.04, 0.5) | 0.96 (0.64, 1.65) | |
| Calcium | Fem | FFQ | Alt | | -0.04 (-0.04, 0.43) | 1.04 (0.59, 1.53) | |
| Calcium | Male | FAO | Alt | | 0.17 (0.17, 0.63) | 0.84 (0.73, 1.88) | |
| Calcium | Male | FFQ | Alt | | 0.09 (0.09, 0.55) | 0.91 (0.67, 1.74) | |
| Fiber | --- | DR | Ref | 0.33 | ---- | --- | Intake of fiber between 21 and 22 grams of fiber per day |
| Fiber | Fem | FAO | Alt | | 0.56 (0.56, 1.17) | 0.57 (0.93, 3.23) | |
| Fiber | Fem | FFQ | Alt | | 0.27 (0.27, 0.88) | 0.76 (0.69, 2.41) | |
| Fiber | Male | FAO | Alt | | 0.55 (0.55, 1.17) | 0.57 (0.92, 3.22) | |
| Fiber | Male | FFQ | Alt | | 0.26 (0.26, 0.88) | 0.77 (0.69, 2.4) | |
| Fruit | --- | DR | Ref | 0.76 | ---- | --- | Intake of fruits between 310 and 340 grams per day |
| Fruit | Fem | FAO | Alt | | 0.36 (0.36, 1.83) | 0.7 (0.31, 6.21) | |
| Fruit | Fem | Sales | Alt | | 0.73 (0.73, 2.19) | 0.48 (0.45, 8.98) | |
| Fruit | Fem | FFQ | Alt | | -0.15 (-0.15, 1.32) | 1.17 (0.19, 3.73) | |
| Fruit | Fem | HBS | Alt | | 0.23 (0.23, 1.71) | 0.79 (0.27, 5.5) | |
| Fruit | Male | FAO | Alt | | 0.32 (0.32, 1.79) | 0.73 (0.3, 5.97) | |
| Fruit | Male | Sales | Alt | | 0.69 (0.69, 2.16) | 0.5 (0.43, 8.64) | |
| Fruit | Male | FFQ | Alt | | -0.19 (-0.19, 1.28) | 1.21 (0.18, 3.58) | |
| Fruit | Male | HBS | Alt | | 0.19 (0.19, 1.66) | 0.83 (0.26, 5.27) | |
| Legumes | --- | DR | Ref | 0.74 | ---- | --- | Intake of legumes between 90 and 100 grams per day |
| Legumes | Fem | FAO | Alt | | -0.08 (-1.49,1.39) | 1.08 (0.22,4) | |
| Legumes | Fem | Sales | Alt | | -0.9 (-2.31,0.56) | 2.47 (0.1,1.75) | |
| Legumes | Fem | FFQ | Alt | | -0.53 (-1.94,0.95) | 1.7 (0.14,2.58) | |
| Legumes | Male | FAO | Alt | | 0.06 (-1.35,1.53) | 0.94 (0.26,4.61) | |
| Legumes | Male | Sales | Alt | | -0.76 (-2.16,0.7) | 2.14 (0.12,2.01) | |
| Legumes | Male | FFQ | Alt | | -0.39 (-1.79,1.09) | 1.47 (0.17,2.98) | |
| Milk | --- | DR | Ref | 1.06 | ---- | --- | Intake of milk between 360 and 500 grams per day |
| Milk | Fem | FAO | Alt | | 0.27 (0.27, 2.57) | 0.76 (0.16, 13.01) | |
| Milk | Fem | Sales | Alt | | 0.01 (0.01, 2.31) | 0.99 (0.13, 10.11) | |
| Milk | Fem | FFQ | Alt | | 0.46 (0.46, 2.78) | 0.63 (0.18, 16.2) | |
| Milk | Fem | HBS | Alt | | -0.61 (-0.61, 1.69) | 1.84 (0.07, 5.4) | |
| Milk | Male | FAO | Alt | | 0.28 (0.28, 2.58) | 0.75 (0.17, 13.17) | |

(Table 1 continues on next page)

(Continued from previous page)

| | | | | | | | |
|---|---|---|---|---|---|---|---|
| Milk | Male | Sales | Alt | | 0.03 (0.03, 2.33) | 0.97 (0.13, 10.23) | |
| Milk | Male | FFQ | Alt | | 0.48 (0.48, 2.8) | 0.62 (0.18, 16.43) | |
| Milk | Male | HBS | Alt | | -0.59 (-0.59, 1.7) | 1.81 (0.07, 5.48) | |
| Nuts and seeds | --- | DR | Ref | | ---- | --- | |
| Nuts and seeds | Fem | FAO | Alt | | 0.49 (0.49, 3.63) | 0.62 (0.06, 37.68) | |
| Nuts and seeds | Fem | FFQ | Alt | | -0.34 (-0.34, 2.76) | 1.41 (0.02, 15.75) | |
| Nuts and seeds | Fem | HBS | Alt | 1.58 | -0.72 (-0.72, 2.42) | 2.06 (0.02, 11.27) | Intake of nuts and seeds between 10 and 19 grams per day |
| Nuts and seeds | Male | FAO | Alt | | 0.6 (0.6, 3.73) | 0.55 (0.07, 41.65) | |
| Nuts and seeds | Male | FFQ | Alt | | -0.23 (-0.23, 2.87) | 1.26 (0.03, 17.58) | |
| Nuts and seeds | Male | HBS | Alt | | -0.62 (-0.62, 2.54) | 1.85 (0.02, 12.66) | |
| Omega-3 | --- | DR | Ref | | ---- | --- | |
| Omega-3 | Male | FAO | Alt | 0.12 | -1.15 (-1.15, -0.92) | 3.16 (0.25, 0.4) | Intake of seafood omega-3 fatty acids between 430 and 470 milligrams per day |
| Omega-3 | Fem | FAO | Alt | | -1.01 (-1.01, -0.78) | 2.75 (0.29, 0.46) | |
| Processed meat | --- | DR | Ref | | ---- | --- | |
| Processed meat | Fem | Sales | Alt | | 0.79 (0.79, 3.14) | 0.46 (0.19, 23.07) | |
| Processed meat | Fem | FFQ | Alt | | -0.3 (-0.3, 2.25) | 1.35 (0.05, 9.49) | |
| Processed meat | Fem | HBS | Alt | 1.21 | -0.46 (-0.46, 1.89) | 1.59 (0.05, 6.63) | No intake of processed meat |
| Processed meat | Male | Sales | Alt | | 0.95 (0.95, 3.3) | 0.39 (0.22, 27.03) | |
| Processed meat | Male | FFQ | Alt | | -0.13 (-0.13, 2.42) | 1.14 (0.06, 11.2) | |
| Processed meat | Male | HBS | Alt | | -0.3 (-0.3, 2.06) | 1.35 (0.06, 7.82) | |
| PUFA | --- | DR | Ref | | ---- | --- | |
| PUFA | Fem | FAO | Alt | | -0.14 (-0.14, 0.14) | 1.15 (0.65, 1.15) | |
| PUFA | Fem | FFQ | Alt | 0.14 | 1.05 (1.05, 1.43) | 0.35 (1.96, 4.18) | Intake of PUFA between 7 and 9% of total daily energy |
| PUFA | Male | FAO | Alt | | -0.18 (-0.18, 0.1) | 1.2 (0.62, 1.1) | |
| PUFA | Male | FFQ | Alt | | 1 (1, 1.38) | 0.37 (1.87, 3.98) | |
| Red meat | --- | DR | Ref | | ---- | --- | |
| Red meat | Fem | FAO | Alt | | 0.89 (0.89, 2.54) | 0.41 (0.45, 12.69) | |
| Red meat | Fem | Sales | Alt | | 1.09 (1.09, 2.74) | 0.34 (0.54, 15.49) | |
| Red meat | Fem | FFQ | Alt | | -0.34 (-0.34, 1.6) | 1.4 (0.11, 4.95) | |
| Red meat | Fem | HBS | Alt | 0.83 | 0.45 (0.45, 2.1) | 0.64 (0.29, 8.18) | No intake of red meat |
| Red meat | Male | FAO | Alt | | 0.89 (0.89, 2.54) | 0.41 (0.45, 12.66) | |
| Red meat | Male | Sales | Alt | | 1.09 (1.09, 2.74) | 0.34 (0.54, 15.43) | |
| Red meat | Male | FFQ | Alt | | -0.34 (-0.34, 1.6) | 1.4 (0.11, 4.94) | |
| Red meat | Male | HBS | Alt | | 0.45 (0.45, 2.1) | 0.64 (0.29, 8.15) | |
| Sodium | --- | Urinary sodium | Ref | | ---- | --- | |
| Sodium | Fem | DR | Alt | | -0.2 (-0.02, 0.85) | 1.02 (0.38, 2.34) | |
| Sodium | Fem | FFQ | Alt | | 0.47 (0.47. 1.29) | 0.63 (0.69, 3.64) | Average 24-hour urinary sodium between 1 and 5 grams per day |
| Sodium | Male | DR | Alt | 0.39 | -0.06 (-0.06, 0.8) | 1.06 (0.38, 2.23) | |
| Sodium | Male | FFQ | Alt | | 0.43 (0.43, 1.26) | 0.65 (0.67, 3.52) | |
| SSBs | --- | DR | Ref | 0.61 | ---- | --- | |
| SSBs | Fem | Sales | Alt | | 0.15 (0.15, 1.43) | 0.86 (0.37, 4.17) | |

(Table 1 continues on next page)

(Continued from previous page)

| | | | | | | | |
|---|---|---|---|---|---|---|---|
| SSBs | Fem | FFQ | Alt | | -0.01 (-0.01, 1.32) | 1.01 (0.3, 3.75) | No intake of beverages with ≥50 kcal per 226.8 gram serving |
| SSBs | Fem | HBS | Alt | | -0.59 (-0.59, 0.68) | 1.8 (0.18, 1.98) | |
| SSBs | Male | Sales | Alt | | 0.35 (0.35, 1.63) | 0.7 (0.45, 5.1) | |
| SSBs | Male | FFQ | Alt | | 0.19 (0.19, 1.53) | 0.83 (0.37, 4.6) | |
| SSBs | Male | HBS | Alt | | -0.39 (-0.39, 0.89) | 1.48 (0.22, 2.43) | |
| Trans fatty acids | --- | DR | Ref | | ---- | --- | |
| Trans fatty acids | Male | Sales | Alt | | -0.23 (-1.27,0.94) | 1.25 (0.28, 2.55) | |
| Trans fatty acids | Fem | Sales | Alt | 0.22 | -0.23 (-1.27,0.94) | 1.25 (0.28, 2.55) | No intake of trans fatty acids |
| Trans fatty acids | Male | FFQ | Alt | | 0.59 (-2.72,4.23) | 0.56 (0.07,68.72) | |
| Trans fatty acids | Fem | FFQ | Alt | | 0.86 (-2.63,4.9) | 0.42 (0.07,134.0) | |
| Vegetables | --- | DR | Ref | | ---- | --- | |
| Vegetables | Fem | FAO | Alt | | 0.12 (0.12, 1.33) | 0.89 (0.31, 3.78) | |
| Vegetables | Fem | Sales | Alt | | 0.62 (0.62, 1.83) | 0.54 (0.51, 6.21) | |
| Vegetables | Fem | FFQ | Alt | | -0.05 (-0.05, 1.16) | 1.05 (0.26, 3.18) | |
| Vegetables | Fem | HBS | Alt | | 0.1 (0.1, 1.31) | 0.91 (0.3, 3.69) | |
| Vegetables | Male | FAO | Alt | 0.64 | 0.16 (0.16, 1.37) | 0.85 (0.32, 3.94) | Intake of vegetables between 280 and 320 grams per day |
| Vegetables | Male | Sales | Alt | | 0.66 (0.66, 1.87) | 0.52 (0.53, 6.49) | |
| Vegetables | Male | FFQ | Alt | | -0.01 (-0.01, 1.2) | 1.01 (0.27, 3.32) | |
| Vegetables | Male | HBS | Alt | | 0.14 (0.14, 1.35) | 0.87 (0.32, 3.85) | |
| Whole grains | --- | DR | Ref | | ---- | --- | |
| Whole grains | Fem | FAO | Alt | | 1.94 (1.94, 3.37) | 0.14 (1.82, 29.05) | |
| Whole grains | Fem | FFQ | Alt | 0.69 | -0.35 (-0.35, 1.37) | 1.42 (0.13, 3.94) | Intake of whole grains between 140 and 160 grams |
| Whole grains | Male | FAO | Alt | | 2.09 (2.09, 3.52) | 0.12 (2.12, 33.76) | |
| Whole grains | Male | FFQ | Alt | | -0.2 (-0.2, 1.52) | 1.22 (0.15, 4.58) | |

**95%CI:** 95% Coefficient Interval; **Alt.:** Alternative; **DR:** 24-hour dietary recall; **FAO:** Food and Agriculture Organization; **FFQ:** Food Frequency Questionnaire; **HBS:** Household Budge Survey; **PUFA:** polyunsaturated fatty acids; **Ref.:** Reference; **SSBs:** sugar-sweetened beverages; **TMREL**: Theoretical minimum risk exposure level. Source: Adapted from the GBD 2019 Risk Factors Collaborators Supplement.
