# Supplementary material for: Burden of non-communicable diseases attributable to dietary risks in Brazil, 1990-2019: an analysis of the Global Burden of Disease Study 2019
Source: Rev Soc Bras Med Trop. 2022 Jan 28;55(Suppl 1):e0282-2021. doi: 10.1590/0037-8682-0282-2021 (PMC9009426; doi:10.1590/0037-8682-0282-2021)
Supplement: Supplementary file 2 [file 1678-9849-rsbmt-55-s01-e0282-2021-supp2.pdf]

SUPPLEMENTARY MATERIAL

This Supplementary Material provides detailed tables with the description of methodological issues and values for the article “Burden of non-communicable diseases attributable to dietary risks in Brazil, 1990–2019: an analysis of the Global Burden of Disease Study 2019”.

SUPPLEMENTARY TABLE 2: Epidemiological evidence to support the causal relationship between dietary risk factors and outcomes.

Dietary risk	Outcome
Calcium	**Neoplasms:** Colon and rectum cancer.
Fiber	**Cardiovascular diseases:** Ischemic heart disease; Ischemic stroke; Intracerebral hemorrhage; Subarachnoid hemorrhage. **Diabetes:** Diabetes mellitus type 2.
Fruit	**Neoplasms:** Esophageal cancer; Tracheal, bronchus, and lung cancer. **Cardiovascular diseases:** Ischemic heart disease; Ischemic stroke; Intracerebral hemorrhage; Subarachnoid hemorrhage. **Diabetes:** Diabetes mellitus type 2.
Legumes	**Cardiovascular diseases:** Ischemic heart disease.
Milk	**Neoplasms:** Colon and rectum cancer.
Nuts and seeds	**Cardiovascular diseases:** Ischemic heart disease. **Diabetes:** Diabetes mellitus type 2.
Seafood omega-3 fatty acids	**Cardiovascular diseases:** Ischemic heart disease.
Processed meat	**Neoplasms:** Colon and rectum cancer **Cardiovascular diseases:** Ischemic heart Disease. **Diabetes:** Diabetes mellitus type 2.
Polyunsaturated fatty acids	**Cardiovascular diseases:** Ischemic heart disease.
Red meat	**Neoplasms:** Breast cancer; Colon and rectum cancer. **Cardiovascular diseases:** Ischemic heart disease; Ischemic stroke; Intracerebral hemorrhage; Subarachnoid hemorrhage. **Diabetes:** Diabetes mellitus type 2.
Sodium	**Neoplasms:** Stomach cancer. **Cardiovascular diseases:** Ischemic heart Disease; Ischemic stroke; Intracerebral hemorrhage; Subarachnoid hemorrhage; Hypertensive heart disease; Rheumatic heart disease; Atrial fibrillation and flutter; Aortic aneurysm; Other cardiomyopathy; Other cardiovascular and circulatory disease. **Kidney diseases:** Chronic kidney disease due to other and unspecified causes; Chronic kidney disease due to hypertension; Chronic kidney disease due to diabetes mellitus type 2; Chronic kidney disease due to glomerulonephritis.
Sugar-sweetened beverages	**Cardiovascular diseases:** Ischemic heart disease. **Diabetes:** Diabetes mellitus type 2.
Trans fatty acids	**Cardiovascular diseases:** Ischemic heart disease.
Vegetables	**Neoplasms:** Esophageal cancer. **Cardiovascular diseases:** Ischemic heart disease; Ischemic stroke; Intracerebral hemorrhage; Subarachnoid hemorrhage.
Whole grains	**Neoplasms:** Colon and rectum cancer. **Cardiovascular diseases:** Ischemic heart disease; Ischemic stroke. **Diabetes:** Diabetes mellitus type 2.

Source: Elaborated by the authors with data from the GBD 2019 Risk Factors Collaborators Supplement.
