# Supplementary material for: Burden of non-communicable diseases attributable to dietary risks in Brazil, 1990-2019: an analysis of the Global Burden of Disease Study 2019
Source: Rev Soc Bras Med Trop. 2022 Jan 28;55(Suppl 1):e0282-2021. doi: 10.1590/0037-8682-0282-2021 (PMC9009426; doi:10.1590/0037-8682-0282-2021)
Supplement: Supplementary file 3 [file 1678-9849-rsbmt-55-s01-e0282-2021-supp3.pdf]

SUPPLEMENTARY MATERIAL

This Supplementary Material provides detailed tables with the description of methodological issues and values for the article “Burden of non-communicable diseases attributable to dietary risks in Brazil, 1990–2019: an analysis of the Global Burden of Disease Study 2019”.

SUPPLEMENTARY TABLE 3: Mean intake of dietary factors among adults aged 25 years or older in Brazil and Federative Units in 2019.

Location	Diet high in processed meat (g/day)	Diet low in polyunsaturated fatty acids (% energy/day)	Diet high in red meat (g/day)	Diet high in sodium (g/day)	Diet high in sugar-sweetened beverages (g/day)	Diet high in trans fatty acids (g/day)	Diet low in vegetables (g/day)	Diet low in calcium (g/day)	Diet low in fiber (g/day)	Diet low in seafood omega-3 fatty acids (% energy/day)	Diet low in fruits (g/day)	Diet low in whole grains (g/day)	Diet low in legumes (g/day)	Diet low in milk (g/day)	Diet low in nuts and seeds (g/day)
**Brazil**	8.18 (7.661-8.734)	0.005 (0.005-0.006)	68.353 (64.136-73.001)	3.546 (3.344-3.743)	111.007 (93.124-130.529)	0.005 (0.005-0.006)	101.525 (97.084-106.392)	0.626 (0.618-0.634)	15.357 (14.943-15.788)	0.086 (0.083-0.089)	202.44 (188.921-215.78)	34.765 (33.913-35.645)	95.703 (90.402-101.514)	173.316 (167.87-178.936)	20.23 (18.905-21.554)
Acre	7.975 (6.658-9.657)	0.005 (0.005-0.006)	67.64 (58.133-78.844)	3.555 (3.257-3.881)	89.94 (52.867-154.991)	0.005 (0.005-0.006)	95.858 (87.067-105.794)	0.554 (0.54-0.569)	14.313 (13.297-15.334)	0.07 (0.064-0.077)	191.697 (164.708-222.244)	32.193 (29.254-35.785)	84.664 (76.112-94.02)	150.096 (139.979-159.572)	15.773 (13.565-18.436)
Alagoas	7.554 (6.396-9.041)	0.005 (0.005-0.006)	57.776 (50.286-66.606)	3.538 (3.263-3.838)	69.065 (41.664-111.288)	0.005 (0.005-0.006)	98.031 (89.042-107.945)	0.508 (0.496-0.521)	13.525 (12.642-14.495)	0.051 (0.047-0.056)	189.806 (165.581-217.651)	31.423 (28.365-34.788)	78.371 (70.701-86.803)	186.583 (174.641-198.988)	13.699 (11.872-15.835)
Amazonas	8.466 (7.037-10.183)	0.005 (0.005-0.006)	72.102 (62.439-82.894)	3.559 (3.254-3.873)	113.211 (65.184-184.398)	0.005 (0.005-0.006)	105.651 (95.74-116.023)	0.611 (0.594-0.629)	15.389 (14.271-16.537)	0.078 (0.072-0.085)	195.563 (168.839-223.301)	33.637 (30.453-37.287)	92.944 (83.711-103.169)	148.604 (138.388-159.02)	18.769 (16.174-21.811)
Amapá	8.235 (6.857-10.164)	0.005 (0.005-0.006)	60.679 (52.103-71.218)	3.551 (3.262-3.877)	98.499 (56.537-170.547)	0.005 (0.005-0.006)	113.237 (102.302-125.636)	0.573 (0.556-0.589)	14.582 (13.491-15.738)	0.068 (0.062-0.074)	213.556 (185.605-244.921)	32.505 (29.155-36.175)	87.178 (78.034-96.969)	135.399 (126.01-144.919)	16.649 (14.161-19.484)
Bahia	7.657 (6.459-8.955)	0.005 (0.005-0.006)	64.176 (56.351-72.581)	3.541 (3.277-3.819)	77.643 (49.615-116.254)	0.005 (0.005-0.006)	92.47 (84.194-100.909)	0.543 (0.529-0.556)	14.272 (13.326-15.215)	0.06 (0.055-0.065)	194.184 (171.233-221.919)	32.417 (29.52-35.454)	83.105 (75.84-90.643)	146.693 (138.293-155.491)	15.354 (13.298-17.648)
Ceará	7.528 (6.408-8.868)	0.005 (0.005-0.006)	60.391 (52.519-69.112)	3.538 (3.28-3.843)	72.843 (45.333-116.172)	0.005 (0.005-0.006)	92.382 (84.419-101.103)	0.523 (0.51-0.536)	13.888 (13.003-14.845)	0.055 (0.051-0.059)	178.641 (156.746-202.285)	31.86 (28.963-34.872)	80.205 (72.948-88.225)	194.447 (183.206-205.732)	14.422 (12.559-16.679)
Distrito Federal	10.113 (8.424-12.033)	0.005 (0.005-0.006)	68.864 (59.533-79.146)	3.537 (3.245-3.861)	266.128 (159.273-422.689)	0.005 (0.005-0.006)	149.048 (135.476-163.424)	0.881 (0.856-0.903)	20.428 (19.057-21.825)	0.179 (0.165-0.195)	264.712 (232.053-299.795)	40.346 (36.369-44.821)	130.765 (118.564-144.072)	229.339 (214.313-244.399)	35.285 (30.334-40.964)
Espírito Santo	8.667 (7.221-10.299)	0.005 (0.005-0.006)	65.204 (56.713-75.143)	3.549 (3.258-3.834)	137.038 (85.18-218.375)	0.005 (0.005-0.006)	113.229 (103.108-124.258)	0.681 (0.663-0.7)	17.072 (15.919-18.351)	0.099 (0.091-0.107)	232.431 (203.58-263.531)	36.229 (32.915-39.685)	102.724 (93.186-112.869)	177.927 (167.43-189.329)	22.821 (19.78-26.389)
Goiás	8.141 (6.797-9.56)	0.005 (0.005-0.006)	67.223 (58.82-76.428)	3.554 (3.273-3.843)	106.563 (64.949-172.29)	0.005 (0.005-0.006)	101.599 (93.051-111.228)	0.614 (0.598-0.63)	15.707 (14.65-16.883)	0.079 (0.073-0.085)	197.832 (172.145-228.616)	34.267 (30.906-37.638)	93.221 (84.986-102.305)	205.068 (192.295-218.445)	19.093 (16.56-21.859)
Maranhão	7.316 (6.194-8.733)	0.005 (0.005-0.006)	62.779 (54.666-72.393)	3.545 (3.261-3.842)	64.214 (38.259-104.749)	0.005 (0.005-0.006)	95.956 (86.512-105.276)	0.49 (0.477-0.503)	13.071 (12.215-13.985)	0.047 (0.043-0.051)	177.914 (156.252-203.351)	30.634 (28.011-33.862)	75.486 (68.179-83.283)	151.079 (141.622-160.344)	12.803 (10.961-14.899)
Minas Gerais	8.035 (6.926-9.406)	0.005 (0.005-0.006)	65.197 (57.586-73.299)	3.536 (3.285-3.788)	103.133 (67.263-155.318)	0.005 (0.005-0.006)	98.68 (91.696-106.495)	0.698 (0.683-0.714)	12.132 (11.356-12.944)	0.082 (0.076-0.088)	205.831 (180.503-231.054)	34.751 (31.707-37.631)	94.17 (85.87-103.303)	184.04 (173.987-194.295)	19.53 (17.159-22.158)
Mato Grosso do Sul	8.128 (6.791-9.638)	0.005 (0.005-0.006)	69.958 (60.558-79.947)	3.553 (3.294-3.842)	113.204 (70.666-183.078)	0.005 (0.005-0.006)	105.048 (95.383-115.521)	0.626 (0.61-0.643)	16.003 (14.91-17.218)	0.083 (0.076-0.09)	209.137 (181.219-239.261)	34.595 (31.606-38.008)	95.204 (86.121-104.682)	203.284 (191.39-215.27)	19.792 (17.063-22.918)
Mato Grosso	8.246 (6.782-9.794)	0.005 (0.005-0.006)	73.251 (64.186-83.654)	3.564 (3.273-3.863)	129.183 (77.458-205.279)	0.005 (0.005-0.006)	105.671 (96.192-115.807)	0.654 (0.636-0.671)	16.473 (15.356-17.619)	0.102 (0.094-0.112)	218.251 (189.912-250.386)	35.166 (31.989-38.639)	98.908 (89.177-110.036)	225.471 (211.158-241.537)	21.278 (18.581-24.322)
Pará	7.801 (6.526-9.34)	0.005 (0.005-0.006)	65.702 (56.898-75.823)	3.56 (3.267-3.858)	80.655 (48.851-127.537)	0.005 (0.005-0.006)	99.023 (90.07-108.886)	0.537 (0.522-0.551)	14.018 (13.085-15.072)	0.058 (0.054-0.064)	215.799 (187.492-245.489)	31.851 (28.849-35.112)	82.327 (73.999-91.005)	134.645 (125.797-143.837)	14.946 (12.708-17.387)
Paraíba	7.5 (6.328-8.986)	0.005 (0.005-0.006)	57.316 (49.825-65.454)	3.536 (3.261-3.825)	69.741 (43.341-115.076)	0.005 (0.005-0.006)	96.22 (87.816-105.852)	0.515 (0.502-0.528)	13.774 (12.885-14.707)	0.053 (0.049-0.057)	186.367 (162.375-213.463)	31.753 (28.862-34.8)	78.994 (71.79-86.802)	181.935 (170.128-194.119)	14.037 (12.207-16.242)
Paraná	8.142 (6.864-9.535)	0.005 (0.005-0.006)	67.727 (59.36-76.924)	3.534 (3.272-3.824)	119.144 (77.368-174.643)	0.005 (0.005-0.006)	104.26 (95.45-114.265)	0.655 (0.638-0.673)	16.631 (15.594-17.767)	0.091 (0.084-0.098)	205.498 (178.269-234.921)	35.619 (32.5-38.841)	98.984 (90.134-108.634)	169.536 (159.911-179.642)	21.31 (18.704-24.37)
Pernambuco	7.748 (6.539-9.111)	0.005 (0.005-0.006)	62.283 (54.331-71.241)	3.531 (3.271-3.816)	79.935 (50.188-126.305)	0.005 (0.005-0.006)	95.903 (86.739-104.339)	0.547 (0.533-0.561)	14.366 (13.459-15.361)	0.061 (0.056-0.066)	189.826 (166.951-215.89)	32.572 (29.949-35.901)	83.655 (75.76-92.018)	179.157 (168.255-190.802)	15.549 (13.538-17.839)
Piauí	7.14 (5.989-8.388)	0.005 (0.005-0.006)	66.755 (58.178-76.043)	3.544 (3.261-3.833)	60.855 (37.556-99.08)	0.005 (0.005-0.006)	93.622 (86.174-102.188)	0.489 (0.475-0.501)	13.233 (12.379-14.104)	0.047 (0.043-0.05)	186.693 (162.449-211.921)	30.945 (28.175-33.867)	75.419 (67.943-83.034)	186.984 (176.374-198.824)	12.832 (11.045-14.653)
Rio de Janeiro	8.636 (7.419-9.983)	0.005 (0.005-0.006)	61.383 (54.053-68.984)	3.53 (3.269-3.773)	133.649 (88.476-197.108)	0.005 (0.005-0.006)	102.532 (94.116-111.576)	0.697 (0.681-0.714)	17.448 (16.359-18.542)	0.103 (0.096-0.112)	206.095 (181.615-233.729)	36.704 (33.503-40.084)	104.775 (95.695-114.569)	156.079 (146.736-166.113)	23.783 (20.947-27.085)
Rio Grande do Norte	7.693 (6.494-9.08)	0.005 (0.005-0.006)	61.263 (53.692-70.359)	3.54 (3.255-3.861)	78.397 (48.147-129.631)	0.005 (0.005-0.006)	91.742 (83.761-100.23)	0.539 (0.526-0.554)	14.213 (13.28-15.177)	0.059 (0.054-0.064)	179.146 (156.339-203.223)	32.319 (29.404-35.45)	82.853 (75.119-91.172)	201.164 (189.557-214.093)	15.215 (13.213-17.485)
Rondônia	8.262 (6.919-9.886)	0.005 (0.005-0.006)	66.815 (57.783-77.011)	3.561 (3.27-3.861)	106.733 (66.17-171.195)	0.005 (0.005-0.006)	104.861 (95.602-115.657)	0.602 (0.585-0.619)	15.408 (14.391-16.486)	0.085 (0.078-0.092)	243.073 (211.019-278.442)	33.693 (30.408-36.969)	91.764 (82.934-102.088)	184.255 (171.823-196.535)	18.454 (15.858-21.292)
Roraima	8.222 (6.783-9.782)	0.005 (0.005-0.006)	64.306 (55.197-74.228)	3.578 (3.272-3.917)	104.053 (58.915-179.354)	0.005 (0.005-0.006)	112.783 (101.918-125.08)	0.582 (0.566-0.598)	14.912 (13.866-16.03)	0.079 (0.072-0.086)	194.438 (166.49-225.766)	32.859 (29.894-36.189)	88.709 (79.789-98.336)	176.012 (163.233-190.741)	17.28 (14.736-20.059)
Rio Grande do Sul	8.115 (6.921-9.35)	0.006 (0.005-0.006)	58.862 (51.417-67.193)	3.539 (3.307-3.812)	121.11 (76.653-190.964)	0.006 (0.005-0.006)	100.076 (91.257-108.69)	0.717 (0.701-0.734)	15.465 (14.501-16.413)	0.096 (0.089-0.103)	213.248 (185.233-242.992)	36.207 (33.062-39.689)	100.992 (92.642-110.116)	168.869 (159.119-178.501)	22.185 (19.498-25.474)
Santa Catarina	7.915 (6.687-9.323)	0.005 (0.005-0.006)	64.122 (56.074-73.322)	3.548 (3.288-3.822)	134.41 (84.621-204.753)	0.005 (0.005-0.006)	108.891 (99.953-119.547)	0.679 (0.661-0.696)	17.048 (15.963-18.248)	0.099 (0.091-0.107)	209.798 (184.225-240.913)	36.028 (32.877-39.569)	102.614 (92.886-113.044)	159.482 (149.543-170.093)	22.776 (19.785-26.322)
Sergipe	7.908 (6.596-9.412)	0.005 (0.005-0.006)	64.915 (56.589-74.782)	3.539 (3.267-3.814)	85.847 (53.394-140.127)	0.005 (0.005-0.006)	100.32 (91.51-109.885)	0.556 (0.541-0.572)	14.484 (13.479-15.507)	0.063 (0.058-0.069)	202.836 (177.156-230.816)	32.637 (29.803-35.637)	84.933 (76.836-94.488)	147.945 (137.728-158.303)	15.927 (13.766-18.531)
São Paulo	8.75 (7.653-9.973)	0.005 (0.005-0.006)	83.27 (74.987-92.705)	3.563 (3.315-3.845)	135.286 (98.458-183.543)	0.005 (0.005-0.006)	104.82 (97.287-113.218)	0.643 (0.629-0.657)	16.769 (15.93-17.676)	0.11 (0.104-0.117)	202.726 (179.875-226.349)	36.946 (34.401-39.623)	107.921 (99.487-117.313)	175.859 (166.766-184.948)	24.997 (22.399-27.989)
Tocantins	7.867 (6.607-9.346)	0.005 (0.005-0.006)	73.268 (63.648-84.128)	3.563 (3.274-3.851)	89.018 (51.845-147.219)	0.005 (0.005-0.006)	103.046 (93.706-113.751)	0.558 (0.543-0.573)	14.529 (13.526-15.521)	0.072 (0.066-0.078)	198.561 (170.695-227.454)	32.505 (29.521-35.64)	85.349 (77.043-94.178)	197.045 (184.836-210.527)	16.09 (13.87-18.5)

Data in parentheses are 95% Uncertainty Intervals (95%UI).
