# Supplementary material for: Burden of non-communicable diseases attributable to dietary risks in Brazil, 1990-2019: an analysis of the Global Burden of Disease Study 2019
Source: Rev Soc Bras Med Trop. 2022 Jan 28;55(Suppl 1):e0282-2021. doi: 10.1590/0037-8682-0282-2021 (PMC9009426; doi:10.1590/0037-8682-0282-2021)
Supplement: Supplementary file 4 [file 1678-9849-rsbmt-55-s01-e0282-2021-supp4.pdf]

SUPPLEMENTARY MATERIAL

This Supplementary Material provides detailed tables with the description of methodological issues and values for the article "Burden of non-communicable diseases attributable to dietary risks in Brazil, 1990–2019: an analysis of the Global Burden of Disease Study 2019".

SUPPLEMENTARY TABLE 4: Number of deaths and disability-adjusted life years (DALYs) due non-communicable diseases attributable to dietary risks for both sexes and all ages in Brazil, 2019.

Dietary risk	Number of deaths				Number of DALYs			
	Cardiovascular diseases	Diabetes mellitus	Neoplasms	Chronic kidney disease	Cardiovascular diseases	Diabetes mellitus	Neoplasms	Chronic kidney disease
Diet high in red meat	28798.19 (17850.79-38972.01)	7581.56 (5318.65-9919.56)	3469.27 (1981.07-5093.99)	-	774916.09 (499637.46-1031949.24)	311302.83 (218863.58-416876.37)	94126.90 (56565.37-133874.05)	-
Diet low in whole grains	26602.70 (11586.79-35500.52)	2773.24 (758.62-4261.02)	3874.32 (1444.89-5212.92)	-	640331.53 (274906.49-853286.33)	106531.92 (26081.43-171494.41)	92100.25 (34346.78-123650.8)	-
Diet high in sodium	26635.61 (1805.46-70381.35)	-	1707.17 (47.86-7057.94)	2471.51 (175.24-6682.99)	600352.65 (38266.03-1589891.74)	-	40184.48 (1135.19-166447.71)	58582.79 (4183.87-156441.60)
Diet low in vegetables	16656.31 (10395.01-22784.53)	-	1044.91 (94.43-2096.74)	-	414184.15 (248835.93-570196.36)	-	26668.72 (2384.46-53397.27)	-
Diet low in fiber	11156.09 (5736.05-17202.71)	1835.01 (679.60-3034.34)	496.32 (197.05-1012.64)	-	280536.81 (141399.81-429202.67)	68607.02 (26416.64-114641.79)	11865.09 (4653.94-23997.92)	-
Diet high in trans fatty acids	12616.69 (1173.55-16849.04)	-	-	-	299051.01 (27318.71-397506.67)	-	-	-
Diet low in fruits	8624.42 (4876.8-12563.48)	1465.31 (481.92-3013.14)	1398.51 (551.19-2618.36)	-	226286.71 (129602.72-332391.54)	55219.82 (18446.81-116335.33)	34074.59 (13221.12-64984.04)	-
Diet high in sugar-sweetened beverages	4820.60 (2731.70-6432.58)	2837.30 (1711.66-3802.27)	-	-	116651.05 (61263.16-157144.93)	116785.04 (69002.38-161955.59)	-	-
Diet low in seafood omega-3 fatty acids	5926.06 (3820.91-7311.71)	-	-	-	136477.65 (87418.21-167614.89)	-	-	-
Diet high in processed meat	2172.06 (370.31-6242.76)	2829.64 (1556.36-3515.61)	492.22 (66.72-856.39)	-	56859.45 (7971.75-163839.80)	122615.6 (67965.74-162412.89)	12788.42 (1636.45-21985.45)	-
Diet low in legumes	3874.55 (558.24-7442.60)	-	-	-	77823.81 (11578.52-151776.21)	-	-	-
Diet low in milk	-	-	3559.80 (1767.53-5262.13)	-	-	-	84751.51 (42474.67-125795.77)	-
Diet low in calcium	-	-	2798.59 (1652.27-4310.90)	-	-	-	63979.46 (37357.80-99349.60)	-
Diet low in nuts and seeds	578.07 (358.03-1182.16)	237.86 (126.79-474.48)	-	-	12185.86 (7762.04-25319.73)	8049.48 (4482.16-15346.45)	-	-
Diet low in polyunsaturated fatty acids	1606.8 (466.71-3534.92)	-	-	-	38814.16 (10087.30-86839.80)	-	-	-

DALYs: Disability-adjusted life years.
