# Supplementary material for: Burden of non-communicable diseases attributable to dietary risks in Brazil, 1990-2019: an analysis of the Global Burden of Disease Study 2019
Source: Rev Soc Bras Med Trop. 2022 Jan 28;55(Suppl 1):e0282-2021. doi: 10.1590/0037-8682-0282-2021 (PMC9009426; doi:10.1590/0037-8682-0282-2021)
Supplement: Supplementary file 5 [file 1678-9849-rsbmt-55-s01-e0282-2021-supp5.pdf]

SUPPLEMENTARY MATERIAL

This Supplementary Material provides detailed tables with the description of methodological issues and values for the article "Burden of non-communicable diseases attributable to dietary risks in Brazil, 1990–2019: an analysis of the Global Burden of Disease Study 2019".

SUPPLEMENTARY TABLE 5: Proportion of deaths and disability-adjusted life years (DALYs) among male and female due non-communicable diseases attributable to dietary risks by sex and age in Brazil, 2019.

Dietary risk<br>Deaths	Sex	25-29 years	30-34 years	35-39 years	40-44 years	45-49 years	50-54 years	55-59 years	60-64 years	65-69 years	70-74 years	75-79 years	80-84 years	85-89 years	90-94 years	+95 years
Diet high in red meat	Male	4.07 (2.55-5.44)	4.98 (3.15-6.74)	5.35 (3.34-7.28)	5.35 (3.28-7.39)	5.59 (3.47-7.66)	5.55 (3.41-7.71)	4.72 (2.89-6.62)	4.75 (2.99-6.55)	4.24 (2.70-5.79)	3.77 (2.47-5.11)	3.10 (1.90-4.29)	2.65 (1.56-3.78)	2.37 (1.20-3.80)	2.30 (0.98-3.79)	2.22 (0.93-3.68)
	Female	3.83 (2.78-4.81)	4.83 (3.54-6.06)	5.51 (3.99-7.08)	5.83 (4.27-7.40)	5.96 (4.24-7.70)	5.68 (3.99-7.29)	5.03 (3.46-6.68)	4.88 (3.31-6.51)	4.46 (3.04-6.01)	4.09 (2.79-5.44)	3.46 (2.23-4.75)	2.99 (1.76-4.22)	2.69 (1.23-4.34)	2.52 (1.09-4.43)	2.45 (1.17-4.25)
Diet low in whole grains	Male	3.24 (1.46-4.12)	4.09 (1.87-5.18)	4.49 (2.05-5.73)	4.31 (1.98-5.55)	4.71 (2.21-6.07)	4.66 (2.20-6.00)	4.26 (2.08-5.49)	3.99 (2.05-5.15)	3.63 (1.89-4.65)	3.29 (1.80-4.19)	2.91 (1.60-3.79)	2.80 (1.60-3.64)	2.68 (1.49-3.66)	2.73 (1.41-3.94)	2.72 (1.28-3.94)
	Female	1.74 (0.97-2.16)	2.21 (1.20-2.79)	2.63 (1.40-3.30)	2.75 (1.47-3.50)	3.27 (1.79-4.18)	3.23 (1.73-4.16)	3.12 (1.71-4.02)	3.21 (1.78-4.14)	3.07 (1.73-3.92)	3.00 (1.70-3.83)	2.79 (1.64-3.56)	2.74 (1.62-3.61)	2.68 (1.47-3.76)	2.63 (1.31-3.85)	2.71 (1.28-4.09)
Diet high in sodium	Male	1.42 (0.07-4.44)	2.02 (0.09-5.70)	2.49 (0.09-6.89)	2.89 (0.10-7.85)	3.30 (0.11-8.92)	3.60 (0.11-10.04)	3.97 (0.11-10.69)	4.27 (0.13-11.06)	4.34 (0.14-11.33)	3.83 (0.12-10.46)	3.59 (0.13-9.88)	3.08 (0.12-8.44)	3.06 (0.11-8.50)	3.11 (0.13-8.79)	3.09 (0.12-9.10)
	Female	0.78 (0.06-2.83)	1.11 (0.06-3.93)	1.36 (0.07-4.58)	1.58 (0.08-5.41)	1.75 (0.08-6.03)	1.74 (0.08-6.30)	1.94 (0.08-6.66)	2.26 (0.09-7.32)	2.46 (0.09-8.01)	2.32 (0.09-7.67)	2.50 (0.10-7.99)	2.41 (0.10-7.84)	2.37 (0.10-7.38)	2.40 (0.10-7.68)	2.52 (0.10-8.09)
Diet low in vegetables	Male	1.94 (1.06-2.70)	2.33 (1.30-3.28)	2.67 (1.47-3.76)	2.57 (1.52-3.61)	2.73 (1.64-3.80)	2.70 (1.65-3.74)	2.44 (1.48-3.39)	2.15 (1.32-2.99)	1.96 (1.20-2.66)	1.70 (1.06-2.32)	1.51 (0.96-2.04)	1.43 (0.9-1.97)	1.35 (0.79-1.94)	1.38 (0.76-1.99)	1.38 (0.74-2.07)
	Female	1.36 (0.64-2.07)	1.65 (0.80-2.49)	2.00 (0.97-2.99)	1.97 (1.00-2.94)	2.09 (1.11-3.01)	1.94 (1.04-2.81)	1.76 (0.99-2.56)	1.66 (0.98-2.36)	1.59 (0.94-2.23)	1.45 (0.89-1.98)	1.36 (0.82-1.87)	1.36 (0.86-1.89)	1.31 (0.73-1.87)	1.31 (0.72-1.94)	1.38 (0.72-2.06)
Diet low in fiber	Male	1.80 (0.88-2.77)	2.11 (1.01-3.3)	2.32 (1.07-3.63)	2.07 (0.96-3.23)	1.98 (0.93-3.24)	1.76 (0.83-2.94)	1.47 (0.79-2.28)	1.25 (0.61-2.10)	1.22 (0.57-2.04)	1.20 (0.55-2.02)	1.18 (0.57-1.86)	1.38 (0.72-2.12)	1.06 (0.32-2.09)	1.01 (0.29-2.00)	0.99 (0.27-2.05)
	Female	1.36 (0.65-2.11)	1.61 (0.76-2.55)	1.86 (0.92-2.89)	1.74 (0.87-2.79)	1.72 (0.81-2.80)	1.45 (0.71-2.31)	1.25 (0.69-1.98)	1.12 (0.54-1.88)	1.13 (0.51-1.82)	1.17 (0.56-1.98)	1.23 (0.61-1.97)	1.43 (0.75-2.22)	1.12 (0.32-2.20)	1.03 (0.28-2.10)	1.04 (0.30-2.09)
Diet high in trans fatty acids	Male	1.2 (0.08-1.77)	1.55 (0.11-2.33)	1.79 (0.13-2.67)	1.74 (0.12-2.57)	1.90 (0.13-2.81)	1.90 (0.14-2.82)	1.72 (0.14-2.52)	1.52 (0.13-2.22)	1.38 (0.12-2.05)	1.21 (0.10-1.78)	1.06 (0.09-1.55)	1.02 (0.09-1.51)	0.99 (0.09-1.49)	1.07 (0.10-1.66)	1.13 (0.10-1.77)
	Female	0.54 (0.04-0.79)	0.74 (0.06-1.10)	0.95 (0.07-1.4)	1.01 (0.08-1.50)	1.24 (0.11-1.83)	1.22 (0.10-1.80)	1.17 (0.10-1.75)	1.19 (0.11-1.75)	1.16 (0.11-1.71)	1.08 (0.11-1.58)	1.02 (0.10-1.51)	1.00 (0.10-1.49)	1.00 (0.09-1.52)	1.06 (0.10-1.63)	1.15 (0.10-1.79)
Diet low in fruits	Male	1.58 (0.79-2.55)	1.80 (0.83-2.94)	2.02 (0.94-3.23)	1.82 (0.77-3.00)	1.73 (0.72-2.92)	1.52 (0.60-2.85)	1.28 (0.49-2.35)	1.09 (0.32-2.15)	1.06 (0.43-1.89)	0.96 (0.36-1.69)	0.90 (0.34-1.60)	0.90 (0.37-1.55)	0.95 (0.24-1.88)	0.90 (0.21-1.77)	0.85 (0.19-1.72)
	Female	1.43 (0.78-2.36)	1.68 (0.92-2.68)	1.95 (1.02-3.17)	1.80 (0.89-3.02)	1.74 (0.84-2.84)	1.47 (0.62-2.53)	1.25 (0.52-2.17)	1.09 (0.38-1.96)	1.07 (0.41-1.94)	0.98 (0.40-1.77)	0.94 (0.40-1.72)	0.96 (0.39-1.64)	1.04 (0.28-2.06)	0.96 (0.25-1.88)	0.96 (0.21-1.95)
Diet high in sugar-sweetened beverages	Male	0.79 (0.29-1.36)	0.94 (0.36-1.54)	0.94 (0.38-1.51)	0.86 (0.38-1.33)	0.95 (0.50-1.42)	0.96 (0.51-1.40)	0.91 (0.51-1.26)	0.87 (0.53-1.18)	0.84 (0.54-1.12)	0.76 (0.47-1.04)	0.70 (0.46-0.95)	0.67 (0.44-0.93)	0.54 (0.28-0.99)	0.55 (0.27-0.98)	0.55 (0.26-1.01)
	Female	0.52 (0.25-0.84)	0.60 (0.26-0.98)	0.61 (0.27-1.02)	0.62 (0.30-1.00)	0.72 (0.36-1.19)	0.74 (0.38-1.17)	0.75 (0.43-1.14)	0.79 (0.46-1.14)	0.79 (0.47-1.11)	0.77 (0.47-1.09)	0.73 (0.45-1.03)	0.69 (0.42-0.96)	0.61 (0.29-1.20)	0.58 (0.26-1.15)	0.58 (0.27-1.19)
Diet low in seafood omega-3 fatty acids	Male	0.55 (0.33-0.72)	0.72 (0.43-0.95)	0.84 (0.50-1.12)	0.83 (0.53-1.09)	0.88 (0.55-1.16)	0.83 (0.55-1.08)	0.74 (0.48-0.96)	0.66 (0.45-0.85)	0.61 (0.41-0.78)	0.54 (0.34-0.70)	0.49 (0.32-0.65)	0.49 (0.32-0.64)	0.57 (0.34-0.85)	0.58 (0.34-0.87)	0.62 (0.34-0.93)
	Female	0.25 (0.16-0.33)	0.35 (0.21-0.46)	0.45 (0.25-0.59)	0.49 (0.32-0.66)	0.58 (0.37-0.75)	0.55 (0.36-0.72)	0.51 (0.34-0.67)	0.52 (0.33-0.66)	0.51 (0.33-0.66)	0.49 (0.32-0.63)	0.46 (0.30-0.60)	0.48 (0.30-0.63)	0.57 (0.33-0.84)	0.58 (0.32-0.86)	0.60 (0.32-0.90)
Diet high in processed meat	Male	0.42 (0.11-1.02)	0.57 (0.18-1.34)	0.63 (0.19-1.48)	0.62 (0.21-1.41)	0.70 (0.26-1.54)	0.70 (0.27-1.52)	0.66 (0.27-1.33)	0.61 (0.28-1.17)	0.55 (0.26-1.05)	0.49 (0.23-0.90)	0.42 (0.20-0.74)	0.32 (0.15-0.59)	0.29 (0.13-0.62)	0.27 (0.11-0.59)	0.27 (0.10-0.59)
	Female	0.37 (0.16-0.69)	0.46 (0.21-0.89)	0.53 (0.22-1.06)	0.57 (0.25-1.10)	0.69 (0.31-1.33)	0.72 (0.35-1.35)	0.74 (0.38-1.33)	0.76 (0.39-1.31)	0.73 (0.37-1.22)	0.67 (0.33-1.16)	0.60 (0.31-1.01)	0.45 (0.21-0.77)	0.40 (0.17-0.78)	0.36 (0.15-0.75)	0.36 (0.13-0.78)
Diet low in legumes	Male	0.45 (0.03-1.36)	0.53 (0.04-1.64)	0.54 (0.03-1.67)	0.49 (0.04-1.59)	0.50 (0.04-1.66)	0.46 (0.04-1.47)	0.42 (0.05-1.38)	0.39 (0.05-1.30)	0.38 (0.04-1.15)	0.38 (0.04-1.13)	0.40 (0.04-1.12)	0.49 (0.05-1.32)	0.71 (0.06-1.70)	0.72 (0.06-1.76)	0.78 (0.05-2.03)
	Female	0.11 (0.01-0.41)	0.14 (0.01-0.46)	0.14 (0.02-0.55)	0.14 (0.02-0.54)	0.16 (0.02-0.68)	0.15 (0.03-0.60)	0.15 (0.03-0.52)	0.16 (0.03-0.57)	0.18 (0.03-0.61)	0.19 (0.03-0.68)	0.21 (0.03-0.72)	0.29 (0.04-0.87)	0.45 (0.04-1.36)	0.47 (0.04-1.43)	0.49 (0.05-1.53)

(Table 5 continues on next page)

(Continued from previous page)

Diet low in milk	Male	0.25 (0.12-0.37)	0.33 (0.17-0.49)	0.34 (0.17-0.51)	0.35 (0.17-0.53)	0.36 (0.18-0.54)	0.36 (0.18-0.55)	0.38 (0.18-0.58)	0.40 (0.2-0.59)	0.38 (0.18-0.57)	0.35 (0.17-0.53)	0.32 (0.15-0.47)	0.28 (0.14-0.42)	0.25 (0.12-0.37)	0.19 (0.09-0.30)	0.14 (0.07-0.23)
	Female	0.30 (0.15-0.46)	0.38 (0.19-0.59)	0.43 (0.20-0.65)	0.46 (0.22-0.70)	0.51 (0.25-0.76)	0.50 (0.25-0.76)	0.50 (0.24-0.74)	0.46 (0.21-0.71)	0.43 (0.20-0.65)	0.39 (0.18-0.58)	0.35 (0.17-0.53)	0.31 (0.15-0.46)	0.27 (0.13-0.41)	0.22 (0.11-0.35)	0.16 (0.07-0.25)
Diet low in calcium	Male	0.21 (0.13-0.33)	0.28 (0.17-0.42)	0.28 (0.17-0.42)	0.28 (0.17-0.42)	0.29 (0.17-0.44)	0.28 (0.16-0.43)	0.30 (0.18-0.45)	0.31 (0.18-0.48)	0.31 (0.18-0.48)	0.31 (0.19-0.46)	0.29 (0.18-0.44)	0.30 (0.19-0.42)	0.24 (0.15-0.35)	0.18 (0.11-0.27)	0.13 (0.07-0.21)
	Female	0.21 (0.11-0.34)	0.26 (0.14-0.42)	0.29 (0.15-0.46)	0.29 (0.16-0.49)	0.32 (0.16-0.53)	0.31 (0.15-0.51)	0.30 (0.16-0.51)	0.28 (0.14-0.49)	0.28 (0.14-0.47)	0.27 (0.14-0.45)	0.27 (0.15-0.42)	0.27 (0.15-0.41)	0.22 (0.12-0.35)	0.17 (0.08-0.28)	0.12 (0.05-0.20)
Diet low in nuts and seeds	Male	0.07 (0.02-0.22)	0.10 (0.03-0.33)	0.08 (0.03-0.27)	0.08 (0.04-0.23)	0.08 (0.05-0.22)	0.08 (0.05-0.22)	0.08 (0.05-0.18)	0.07 (0.05-0.16)	0.08 (0.05-0.19)	0.08 (0.05-0.18)	0.08 (0.05-0.19)	0.10 (0.04-0.25)	0.10 (0.04-0.32)	0.10 (0.04-0.36)	0.09 (0.04-0.29)
	Female	0.03 (0.01-0.09)	0.04 (0.02-0.11)	0.04 (0.02-0.13)	0.04 (0.03-0.12)	0.05 (0.03-0.12)	0.05 (0.04-0.11)	0.05 (0.04-0.12)	0.06 (0.05-0.13)	0.07 (0.05-0.13)	0.07 (0.05-0.16)	0.07 (0.05-0.16)	0.09 (0.05-0.22)	0.09 (0.04-0.28)	0.08 (0.04-0.28)	0.08 (0.04-0.28)
Diet low in polyunsaturated fatty acids	Male	0.17 (0.03-0.45)	0.22 (0.04-0.58)	0.25 (0.04-0.65)	0.23 (0.05-0.57)	0.24 (0.05-0.64)	0.24 (0.05-0.59)	0.21 (0.05-0.49)	0.19 (0.05-0.46)	0.17 (0.05-0.42)	0.15 (0.05-0.35)	0.14 (0.04-0.31)	0.13 (0.04-0.32)	0.11 (0.04-0.26)	0.11 (0.04-0.28)	0.11 (0.04-0.30)
	Female	0.09 (0.01-0.22)	0.12 (0.02-0.30)	0.15 (0.02-0.37)	0.15 (0.03-0.37)	0.18 (0.03-0.46)	0.17 (0.03-0.40)	0.16 (0.04-0.38)	0.16 (0.04-0.38)	0.15 (0.04-0.37)	0.15 (0.04-0.34)	0.14 (0.04-0.32)	0.14 (0.04-0.32)	0.12 (0.04-0.31)	0.12 (0.04-0.30)	0.13 (0.04-0.30)
**Dietary risk**	**Sex**	**25-29 years**	**30-34 years**	**35-39 years**	**40-44 years**	**45-49 years**	**50-54 years**	**55-59 years**	**60-64 years**	**65-69 years**	**70-74 years**	**75-79 years**	**80-84 years**	**85-89 years**	**90-94 years**	**+95 years**
**DALYs**																
Diet high in red meat	Male	1.26 (0.82-1.74)	1.87 (1.25-2.58)	2.47 (1.58-3.35)	3.01 (1.94-4.14)	3.65 (2.38-5.01)	4.07 (2.60-5.57)	3.74 (2.39-5.13)	3.96 (2.60-5.39)	3.67 (2.46-4.94)	3.36 (2.26-4.46)	2.82 (1.78-3.85)	2.42 (1.46-3.42)	2.16 (1.13-3.41)	2.06 (0.93-3.35)	1.95 (0.85-3.13)
	Female	0.79 (0.56-1.04)	1.23 (0.87-1.61)	1.75 (1.25-2.32)	2.31 (1.70-3.03)	2.89 (2.06-3.78)	3.25 (2.34-4.22)	3.26 (2.32-4.24)	3.46 (2.46-4.59)	3.38 (2.40-4.46)	3.26 (2.26-4.25)	2.86 (1.89-3.91)	2.53 (1.54-3.51)	2.29 (1.11-3.63)	2.15 (0.98-3.69)	2.07 (1.03-3.52)
Diet low in whole grains	Male	0.90 (0.42-1.20)	1.40 (0.68-1.87)	1.90 (0.91-2.52)	2.23 (1.06-2.93)	2.85 (1.40-3.72)	3.16 (1.57-4.14)	3.12 (1.58-4.01)	3.07 (1.66-3.99)	2.90 (1.60-3.73)	2.71 (1.52-3.48)	2.46 (1.40-3.19)	2.38 (1.40-3.09)	2.29 (1.30-3.12)	2.30 (1.23-3.20)	2.27 (1.13-3.26)
	Female	0.32 (0.20-0.43)	0.51 (0.31-0.67)	0.77 (0.44-1.00)	1.01 (0.58-1.33)	1.45 (0.83-1.9)	1.68 (0.98-2.17)	1.82 (1.09-2.37)	2.03 (1.23-2.65)	2.09 (1.25-2.72)	2.16 (1.31-2.79)	2.10 (1.27-2.69)	2.12 (1.29-2.77)	2.11 (1.20-2.96)	2.09 (1.10-2.99)	2.17 (1.07-3.25)
Diet high in sodium	Male	0.42 (0.02-1.28)	0.71 (0.03-2.10)	1.05 (0.04-2.89)	1.48 (0.05-4.06)	1.96 (0.06-5.22)	2.39 (0.07-6.71)	2.82 (0.08-7.51)	3.18 (0.10-8.21)	3.34 (0.11-8.74)	3.05 (0.09-8.37)	2.93 (0.10-8.11)	2.56 (0.10-7.01)	2.58 (0.09-7.29)	2.60 (0.11-7.29)	2.59 (0.10-7.51)
	Female	0.16 (0.01-0.59)	0.27 (0.02-0.92)	0.40 (0.02-1.36)	0.57 (0.03-1.96)	0.76 (0.03-2.61)	0.87 (0.04-3.10)	1.08 (0.04-3.73)	1.38 (0.05-4.47)	1.61 (0.06-5.21)	1.62 (0.06-5.39)	1.83 (0.07-5.82)	1.82 (0.08-5.97)	1.85 (0.08-5.75)	1.91 (0.08-6.13)	2.04 (0.08-6.50)
Diet low in vegetables	Male	0.53 (0.29-0.76)	0.78 (0.44-1.10)	1.10 (0.61-1.58)	1.28 (0.73-1.83)	1.59 (0.94-2.26)	1.76 (1.04-2.45)	1.70 (1.03-2.37)	1.57 (0.98-2.20)	1.47 (0.89-2.03)	1.32 (0.84-1.81)	1.20 (0.75-1.63)	1.17 (0.73-1.60)	1.11 (0.64-1.58)	1.12 (0.62-1.62)	1.12 (0.61-1.66)
	Female	0.25 (0.11-0.39)	0.37 (0.17-0.58)	0.56 (0.27-0.86)	0.68 (0.33-1.06)	0.87 (0.45-1.27)	0.94 (0.50-1.39)	0.96 (0.52-1.38)	0.98 (0.56-1.41)	1.01 (0.58-1.43)	0.98 (0.60-1.34)	0.96 (0.58-1.34)	1.00 (0.63-1.39)	1.00 (0.56-1.43)	1.01 (0.58-1.49)	1.09 (0.58-1.64)
Diet low in fiber	Male	0.52 (0.25-0.85)	0.75 (0.37-1.21)	1.01 (0.48-1.62)	1.11 (0.53-1.75)	1.24 (0.60-2.04)	1.24 (0.60-2.03)	1.12 (0.62-1.75)	1.01 (0.50-1.66)	1.02 (0.49-1.69)	1.02 (0.49-1.68)	1.03 (0.52-1.61)	1.20 (0.64-1.83)	0.93 (0.28-1.80)	0.87 (0.26-1.70)	0.84 (0.25-1.73)
	Female	0.27 (0.12-0.43)	0.40 (0.18-0.63)	0.57 (0.28-0.91)	0.67 (0.33-1.08)	0.80 (0.39-1.31)	0.80 (0.40-1.27)	0.78 (0.45-1.23)	0.77 (0.38-1.26)	0.83 (0.40-1.34)	0.90 (0.45-1.48)	0.97 (0.49-1.53)	1.15 (0.62-1.76)	0.92 (0.27-1.79)	0.85 (0.24-1.68)	0.85 (0.26-1.71)
Diet high in trans fatty acids	Male	0.31 (0.02-0.49)	0.50 (0.03-0.80)	0.72 (0.05-1.11)	0.85 (0.06-1.31)	1.10 (0.08-1.66)	1.23 (0.09-1.86)	1.19 (0.10-1.79)	1.11 (0.09-1.65)	1.04 (0.09-1.57)	0.94 (0.08-1.39)	0.85 (0.07-1.24)	0.84 (0.07-1.24)	0.81 (0.07-1.24)	0.87 (0.08-1.33)	0.92 (0.08-1.42)
	Female	0.08 (0.01-0.13)	0.15 (0.01-0.24)	0.24 (0.02-0.39)	0.33 (0.03-0.51)	0.50 (0.04-0.77)	0.57 (0.05-0.88)	0.61 (0.05-0.95)	0.68 (0.06-1.03)	0.71 (0.07-1.07)	0.71 (0.07-1.08)	0.71 (0.07-1.07)	0.72 (0.07-1.09)	0.75 (0.07-1.15)	0.81 (0.08-1.23)	0.90 (0.08-1.41)
Diet low in fruits	Male	0.47 (0.23-0.75)	0.65 (0.31-1.07)	0.90 (0.41-1.47)	0.98 (0.41-1.62)	1.08 (0.46-1.84)	1.05 (0.40-1.98)	0.96 (0.36-1.74)	0.86 (0.25-1.64)	0.86 (0.34-1.57)	0.80 (0.30-1.44)	0.77 (0.29-1.42)	0.78 (0.31-1.35)	0.83 (0.20-1.65)	0.78 (0.18-1.53)	0.73 (0.16-1.48)
	Female	0.29 (0.15-0.48)	0.42 (0.22-0.68)	0.61 (0.30-1.00)	0.69 (0.34-1.18)	0.81 (0.37-1.35)	0.80 (0.33-1.38)	0.76 (0.31-1.34)	0.73 (0.24-1.30)	0.76 (0.30-1.41)	0.74 (0.29-1.37)	0.74 (0.31-1.36)	0.78 (0.30-1.35)	0.87 (0.23-1.70)	0.80 (0.20-1.56)	0.80 (0.17-1.59)
Diet high in sugar-sweetened beverages	Male	0.27 (0.12-0.44)	0.39 (0.18-0.62)	0.48 (0.24-0.74)	0.54 (0.29-0.81)	0.69 (0.39-1.01)	0.77 (0.45-1.13)	0.79 (0.49-1.10)	0.79 (0.50-1.08)	0.79 (0.53-1.06)	0.73 (0.47-0.99)	0.69 (0.46-0.93)	0.65 (0.44-0.88)	0.53 (0.29-0.96)	0.53 (0.27-0.92)	0.51 (0.25-0.93)
	Female	0.13 (0.07-0.21)	0.19 (0.10-0.30)	0.25 (0.13-0.41)	0.31 (0.18-0.50)	0.44 (0.24-0.70)	0.52 (0.30-0.82)	0.58 (0.34-0.87)	0.64 (0.38-0.93)	0.67 (0.41-0.95)	0.68 (0.40-0.97)	0.65 (0.40-0.91)	0.61 (0.37-0.86)	0.55 (0.27-1.12)	0.52 (0.24-1.04)	0.51 (0.24-1.04)
Diet low in seafood omega-3 fatty acids	Male	0.14 (0.08-0.21)	0.23 (0.13-0.33)	0.34 (0.19-0.47)	0.41 (0.25-0.55)	0.51 (0.31-0.69)	0.54 (0.35-0.72)	0.52 (0.32-0.68)	0.48 (0.32-0.63)	0.46 (0.31-0.59)	0.42 (0.26-0.54)	0.39 (0.25-0.52)	0.40 (0.25-0.53)	0.47 (0.28-0.70)	0.48 (0.28-0.70)	0.50 (0.28-0.76)
	Female	0.04 (0.02-0.06)	0.07 (0.04-0.10)	0.12 (0.06-0.16)	0.16 (0.10-0.23)	0.23 (0.14-0.32)	0.26 (0.16-0.35)	0.27 (0.17-0.36)	0.30 (0.19-0.39)	0.31 (0.19-0.41)	0.32 (0.21-0.43)	0.32 (0.20-0.43)	0.35 (0.22-0.46)	0.43 (0.25-0.62)	0.44 (0.25-0.65)	0.47 (0.26-0.70)

(Table 5 continues on next page)

(Continued from previous page)

Diet high in processed meat	Male	0.16 (0.06-0.32)	0.25 (0.10-0.49)	0.35 (0.15-0.69)	0.43 (0.19-0.83)	0.55 (0.24-1.08)	0.61 (0.29-1.17)	0.62 (0.28-1.12)	0.61 (0.30-1.05)	0.58 (0.28-0.98)	0.52 (0.26-0.86)	0.45 (0.21-0.74)	0.34 (0.16-0.58)	0.30 (0.13-0.62)	0.28 (0.12-0.56)	0.26 (0.11-0.54)
	Female	0.10 (0.05-0.16)	0.15 (0.08-0.25)	0.23 (0.12-0.39)	0.31 (0.16-0.52)	0.45 (0.23-0.73)	0.54 (0.29-0.87)	0.62 (0.33-1.00)	0.67 (0.37-1.02)	0.67 (0.35-1.04)	0.63 (0.32-1.02)	0.57 (0.30-0.91)	0.43 (0.20-0.70)	0.38 (0.16-0.72)	0.34 (0.15-0.67)	0.32 (0.13-0.68)
Diet low in legumes	Male	0.12 (0.01-0.35)	0.17 (0.01-0.52)	0.21 (0.01-0.67)	0.24 (0.02-0.76)	0.29 (0.03-0.96)	0.30 (0.03-0.95)	0.29 (0.03-0.96)	0.28 (0.03-0.92)	0.29 (0.03-0.86)	0.30 (0.03-0.87)	0.32 (0.03-0.89)	0.40 (0.04-1.08)	0.58 (0.04-1.41)	0.59 (0.05-1.42)	0.63 (0.05-1.65)
	Female	0.02 (0.00-0.06)	0.03 (0.00-0.09)	0.04 (0.00-0.14)	0.05 (0.01-0.18)	0.07 (0.01-0.27)	0.07 (0.01-0.27)	0.08 (0.01-0.28)	0.09 (0.02-0.32)	0.11 (0.02-0.38)	0.13 (0.02-0.44)	0.15 (0.02-0.5)	0.21 (0.02-0.64)	0.34 (0.03-1.04)	0.36 (0.03-1.07)	0.38 (0.04-1.16)
Diet low in milk	Male	0.06 (0.03-0.10)	0.11 (0.05-0.16)	0.14 (0.07-0.21)	0.17 (0.09-0.26)	0.21 (0.10-0.31)	0.23 (0.12-0.35)	0.26 (0.13-0.40)	0.28 (0.14-0.43)	0.28 (0.14-0.42)	0.27 (0.13-0.40)	0.25 (0.11-0.36)	0.22 (0.11-0.32)	0.20 (0.10-0.30)	0.15 (0.07-0.23)	0.11 (0.05-0.18)
	Female	0.05 (0.02-0.08)	0.08 (0.04-0.12)	0.11 (0.05-0.17)	0.15 (0.07-0.23)	0.20 (0.10-0.32)	0.24 (0.12-0.37)	0.26 (0.13-0.40)	0.26 (0.12-0.41)	0.26 (0.12-0.41)	0.25 (0.12-0.38)	0.24 (0.12-0.37)	0.22 (0.11-0.33)	0.20 (0.10-0.30)	0.17 (0.08-0.26)	0.12 (0.06-0.19)
Diet low in calcium	Male	0.06 (0.03-0.09)	0.09 (0.05-0.14)	0.11 (0.07-0.17)	0.14 (0.08-0.21)	0.16 (0.10-0.25)	0.18 (0.11-0.28)	0.20 (0.12-0.31)	0.22 (0.13-0.34)	0.23 (0.14-0.36)	0.23 (0.14-0.34)	0.23 (0.14-0.33)	0.23 (0.15-0.33)	0.19 (0.12-0.28)	0.14 (0.08-0.21)	0.11 (0.06-0.17)
	Female	0.03 (0.02-0.05)	0.05 (0.03-0.09)	0.07 (0.04-0.13)	0.10 (0.05-0.16)	0.13 (0.06-0.22)	0.14 (0.07-0.24)	0.16 (0.08-0.27)	0.16 (0.08-0.28)	0.17 (0.09-0.28)	0.18 (0.09-0.29)	0.18 (0.10-0.3)	0.19 (0.11-0.29)	0.16 (0.09-0.26)	0.13 (0.06-0.21)	0.09 (0.04-0.16)
Diet low in nuts and seeds	Male	0.02 (0.01-0.06)	0.04 (0.01-0.12)	0.04 (0.02-0.12)	0.05 (0.02-0.13)	0.05 (0.03-0.14)	0.06 (0.04-0.16)	0.06 (0.04-0.15)	0.06 (0.04-0.13)	0.07 (0.05-0.16)	0.07 (0.04-0.16)	0.07 (0.04-0.17)	0.09 (0.04-0.24)	0.09 (0.04-0.29)	0.09 (0.04-0.31)	0.08 (0.03-0.25)
	Female	0.01 (0-00.020)	0.01 (0.00-0.03)	0.01 (0.01-0.04)	0.02 (0.01-0.05)	0.03 (0.02-0.07)	0.03 (0.02-0.07)	0.04 (0.03-0.08)	0.05 (0.03-0.10)	0.05 (0.04-0.10)	0.06 (0.04-0.12)	0.06 (0.04-0.13)	0.07 (0.04-0.19)	0.08 (0.04-0.24)	0.07 (0.03-0.22)	0.07 (0.03-0.22)
Diet low in polyunsaturated fatty acids	Male	0.05 (0.01-0.12)	0.07 (0.01-0.19)	0.10 (0.02-0.26)	0.11 (0.02-0.28)	0.14 (0.03-0.36)	0.15 (0.03-0.39)	0.14 (0.04-0.35)	0.13 (0.04-0.34)	0.13 (0.04-0.31)	0.12 (0.04-0.27)	0.11 (0.04-0.25)	0.11 (0.03-0.26)	0.09 (0.03-0.21)	0.09 (0.03-0.23)	0.09 (0.03-0.24)
	Female	0.01 (0.00-0.04)	0.02 (0.00-0.06)	0.04 (0.01-0.10)	0.05 (0.01-0.13)	0.07 (0.01-0.19)	0.08 (0.02-0.19)	0.08 (0.02-0.20)	0.09 (0.02-0.22)	0.10 (0.03-0.23)	0.10 (0.03-0.23)	0.10 (0.03-0.22)	0.10 (0.03-0.24)	0.09 (0.03-0.23)	0.09 (0.03-0.22)	0.10 (0.03-0.23)

DALYs: Disability-adjusted life years.
Data in parenthesis are 95% Uncertain Intervals (95%UI).
