# Supplementary material for: Burden of non-communicable diseases attributable to dietary risks in Brazil, 1990-2019: an analysis of the Global Burden of Disease Study 2019
Source: Rev Soc Bras Med Trop. 2022 Jan 28;55(Suppl 1):e0282-2021. doi: 10.1590/0037-8682-0282-2021 (PMC9009426; doi:10.1590/0037-8682-0282-2021)
Supplement: Supplementary file 6 [file 1678-9849-rsbmt-55-s01-e0282-2021-supp6.pdf]

SUPPLEMENTARY MATERIAL

This Supplementary Material provides detailed tables with the description of methodological issues and values for the article “Burden of non-communicable diseases attributable to dietary risks in Brazil, 1990–2019: an analysis of the Global Burden of Disease Study 2019”.

SUPPLEMENTARY TABLE 6: Proportion of disability-adjusted life years (DALYs) due non-communicable diseases attributable to dietary risks for both sexes and all ages in Brazil its 27 Federative Units, 2019.

Location	Diet low in fruits	Diet low in vegetables	Diet low in legumes	Diet low in whole grains	Diet low in nuts and seeds	Diet low in milk	Diet high in red meat	Diet high in processed meat	Diet high in sugar sweetened beverages	Diet low in fiber	Diet low in calcium	Diet in low seafood omega-3 fatty	Diet low in polyunsaturated fatty acids	Diet high in trans fatty acids	Diet high in sodium	Dietary risks
Brazil	0.68 (0.43-0.95)	0.95 (0.58-1.31)	0.17 (0.02-0.33)	1.80 (1.00-2.32)	0.04 (0.03-0.08)	0.18 (0.09-0.27)	2.54 (1.89-3.22)	0.41 (0.23-0.65)	0.50 (0.34-0.64)	0.78 (0.45-1.14)	0.14 (0.08-0.21)	0.29 (0.19-0.38)	0.08 (0.02-0.19)	0.64 (0.06-0.89)	1.51 (0.09-4.08)	8.36 (6.75-10.53)
Acre	0.56 (0.35-0.84)	0.67 (0.39-0.94)	0.17 (0.02-0.35)	1.25 (0.70-1.64)	0.04 (0.02-0.09)	0.11 (0.05-0.17)	1.87 (1.40-2.35)	0.30 (0.16-0.47)	0.36 (0.24-0.47)	0.63 (0.35-0.94)	0.10 (0.06-0.14)	0.20 (0.12-0.26)	0.07 (0.01-0.17)	0.42 (0.03-0.59)	1.14 (0.06-3.18)	6.15 (4.86-7.98)
Alagoas	0.90 (0.57-1.33)	1.05 (0.63-1.46)	0.35 (0.04-0.68)	2.01 (1.11-2.59)	0.09 (0.04-0.19)	0.11 (0.05-0.17)	2.75 (1.98-3.50)	0.48 (0.25-0.74)	0.58 (0.41-0.75)	1.12 (0.65-1.63)	0.12 (0.08-0.17)	0.34 (0.2-0.45)	0.14 (0.03-0.33)	0.68 (0.06-0.96)	1.63 (0.09-4.47)	9.57 (7.72-12.07)
Amapá	0.50 (0.29-0.74)	0.63 (0.36-0.90)	0.14 (0.02-0.3)	1.23 (0.68-1.61)	0.03 (0.02-0.07)	0.10 (0.05-0.15)	1.78 (1.31-2.29)	0.31 (0.17-0.49)	0.36 (0.24-0.48)	0.59 (0.31-0.89)	0.08 (0.05-0.12)	0.20 (0.13-0.27)	0.06 (0.01-0.16)	0.42 (0.04-0.6)	1.12 (0.06-3.14)	5.96 (4.64-7.8)
Amazonas	0.51 (0.31-0.77)	0.59 (0.34-0.84)	0.10 (0.01-0.24)	1.18 (0.69-1.56)	0.03 (0.02-0.06)	0.13 (0.06-0.19)	1.96 (1.49-2.48)	0.34 (0.2-0.51)	0.38 (0.25-0.51)	0.50 (0.28-0.77)	0.09 (0.05-0.15)	0.18 (0.11-0.24)	0.05 (0.01-0.12)	0.38 (0.03-0.55)	1.10 (0.06-3.1)	6.00 (4.69-7.79)
Bahia	0.70 (0.42-1.07)	0.98 (0.59-1.37)	0.24 (0.03-0.5)	1.74 (0.98-2.29)	0.06 (0.03-0.13)	0.16 (0.08-0.24)	2.55 (1.89-3.23)	0.41 (0.23-0.65)	0.49 (0.33-0.63)	0.86 (0.48-1.28)	0.14 (0.09-0.21)	0.29 (0.18-0.38)	0.10 (0.02-0.24)	0.58 (0.05-0.82)	1.51 (0.08-4.14)	8.43 (6.74-10.77)
Ceará	0.77 (0.46-1.11)	0.95 (0.58-1.34)	0.29 (0.03-0.59)	1.71 (0.93-2.26)	0.06 (0.03-0.14)	0.11 (0.05-0.18)	2.17 (1.56-2.77)	0.35 (0.19-0.57)	0.44 (0.31-0.58)	0.88 (0.48-1.31)	0.14 (0.09-0.20)	0.30 (0.17-0.39)	0.12 (0.02-0.27)	0.6 (0.05-0.85)	1.39 (0.07-3.82)	7.97 (6.35-10.09)
Distrito Federal	0.25 (0.15-0.40)	0.44 (0.23-0.66)	0.02 (0.01-0.05)	1.23 (0.74-1.61)	0.02 (0.02-0.02)	0.13 (0.05-0.21)	2.12 (1.58-2.67)	0.41 (0.24-0.62)	0.58 (0.35-0.82)	0.22 (0.11-0.36)	0.07 (0.04-0.12)	0.16 (0.1-0.22)	0.02 (0.01-0.05)	0.43 (0.04-0.62)	1.11 (0.06-3.05)	5.96 (4.67-7.76)
Espírito Santo	0.53 (0.31-0.83)	0.94 (0.56-1.33)	0.11 (0.02-0.27)	1.77 (0.98-2.32)	0.03 (0.03-0.05)	0.17 (0.08-0.25)	2.48 (1.83-3.19)	0.43 (0.23-0.68)	0.53 (0.33-0.75)	0.53 (0.28-0.84)	0.12 (0.06-0.2)	0.28 (0.19-0.36)	0.06 (0.02-0.15)	0.65 (0.06-0.91)	1.6 (0.09-4.42)	8.18 (6.45-10.7)
Goiás	0.70 (0.43-1.03)	0.93 (0.56-1.31)	0.16 (0.02-0.38)	1.80 (0.97-2.36)	0.04 (0.02-0.07)	0.15 (0.07-0.24)	2.44 (1.76-3.18)	0.39 (0.21-0.65)	0.47 (0.30-0.66)	0.65 (0.34-1.00)	0.13 (0.08-0.21)	0.30 (0.20-0.39)	0.09 (0.02-0.20)	0.66 (0.06-0.93)	1.45 (0.08-3.98)	8.09 (6.46-10.26)
Maranhão	0.95 (0.59-1.4)	1.12 (0.66-1.57)	0.44 (0.05-0.85)	2.08 (1.10-2.77)	0.10 (0.04-0.23)	0.11 (0.06-0.17)	2.68 (1.93-3.48)	0.42 (0.22-0.7)	0.56 (0.40-0.71)	1.20 (0.68-1.79)	0.12 (0.08-0.16)	0.38 (0.21-0.51)	0.17 (0.03-0.4)	0.74 (0.06-1.05)	1.6 (0.08-4.39)	9.60 (7.61-12.07)
Mato Grosso	0.53 (0.32-0.79)	0.81 (0.48-1.13)	0.11 (0.02-0.27)	1.59 (0.89-2.06)	0.03 (0.02-0.06)	0.11 (0.05-0.18)	2.47 (1.84-3.11)	0.41 (0.22-0.64)	0.52 (0.33-0.71)	0.54 (0.28-0.84)	0.10 (0.05-0.16)	0.24 (0.16-0.32)	0.06 (0.02-0.14)	0.57 (0.05-0.79)	1.34 (0.07-3.61)	7.51 (5.97-9.57)
Mato Grosso do Sul	0.64 (0.37-0.98)	0.95 (0.57-1.35)	0.16 (0.02-0.37)	1.86 (0.99-2.43)	0.04 (0.03-0.07)	0.14 (0.07-0.23)	2.6 (1.9-3.35)	0.41 (0.22-0.67)	0.53 (0.34-0.70)	0.65 (0.34-1.02)	0.13 (0.07-0.21)	0.31 (0.21-0.40)	0.08 (0.02-0.2)	0.69 (0.06-0.98)	1.53 (0.08-4.12)	8.42 (6.70-10.76)
Minas Gerais	0.62 (0.37-0.93)	0.91 (0.56-1.26)	0.13 (0.02-0.29)	1.60 (0.90-2.08)	0.03 (0.02-0.06)	0.17 (0.08-0.26)	2.33 (1.73-2.99)	0.36 (0.19-0.58)	0.42 (0.27-0.56)	1.28 (0.82-1.78)	0.11 (0.06-0.18)	0.26 (0.17-0.33)	0.07 (0.02-0.16)	0.56 (0.05-0.79)	1.40 (0.07-3.83)	7.82 (6.24-9.93)
Pará	0.53 (0.32-0.8)	0.83 (0.49-1.16)	0.23 (0.03-0.49)	1.58 (0.87-2.07)	0.05 (0.02-0.12)	0.13 (0.07-0.19)	2.32 (1.76-2.97)	0.37 (0.2-0.58)	0.45 (0.31-0.59)	0.80 (0.43-1.19)	0.11 (0.07-0.16)	0.26 (0.16-0.35)	0.10 (0.02-0.23)	0.53 (0.04-0.76)	1.32 (0.07-3.62)	7.47 (5.90-9.65)
Paraíba	0.87 (0.54-1.24)	1.04 (0.62-1.43)	0.36 (0.04-0.72)	2.01 (1.07-2.61)	0.08 (0.04-0.18)	0.12 (0.05-0.18)	2.44 (1.71-3.18)	0.45 (0.24-0.71)	0.57 (0.40-0.74)	1.04 (0.58-1.53)	0.13 (0.08-0.18)	0.36 (0.2-0.47)	0.14 (0.03-0.33)	0.71 (0.06-1.00)	1.54 (0.09-4.13)	9.13 (7.38-11.44)
Paraná	0.67 (0.40-1.01)	0.92 (0.56-1.29)	0.12 (0.02-0.29)	1.79 (1.02-2.29)	0.03 (0.03-0.06)	0.21 (0.10-0.32)	2.51 (1.82-3.21)	0.42 (0.23-0.67)	0.53 (0.35-0.70)	0.57 (0.29-0.90)	0.15 (0.08-0.24)	0.28 (0.19-0.36)	0.07 (0.02-0.16)	0.62 (0.05-0.87)	1.52 (0.08-4.21)	8.26 (6.59-10.59)
Pernambuco	0.83 (0.5-1.23)	1.11 (0.67-1.55)	0.31 (0.03-0.66)	2.11 (1.12-2.75)	0.07 (0.03-0.15)	0.13 (0.06-0.19)	2.69 (1.9-3.46)	0.45 (0.25-0.74)	0.56 (0.39-0.74)	0.97 (0.52-1.47)	0.12 (0.08-0.18)	0.38 (0.23-0.49)	0.14 (0.03-0.32)	0.78 (0.07-1.08)	1.59 (0.09-4.34)	9.41 (7.69-11.8)
Piauí	0.84 (0.51-1.20)	0.98 (0.59-1.33)	0.38 (0.04-0.74)	1.83 (1.00-2.37)	0.09 (0.03-0.20)	0.10 (0.05-0.16)	2.65 (1.99-3.39)	0.39 (0.21-0.61)	0.51 (0.36-0.64)	1.06 (0.59-1.54)	0.13 (0.09-0.19)	0.32 (0.17-0.42)	0.14 (0.02-0.33)	0.62 (0.05-0.87)	1.49 (0.07-4.12)	8.84 (7.11-11.12)
Rio de Janeiro	0.76 (0.46-1.12)	1.12 (0.68-1.55)	0.11 (0.02-0.25)	2.16 (1.19-2.81)	0.04 (0.03-0.05)	0.26 (0.13-0.38)	2.83 (2.02-3.71)	0.52 (0.28-0.83)	0.62 (0.38-0.87)	0.58 (0.31-0.91)	0.15 (0.08-0.25)	0.35 (0.23-0.46)	0.08 (0.03-0.17)	0.81 (0.07-1.13)	1.73 (0.08-4.68)	9.58 (7.69-12.18)
Rio Grande do Norte	0.77 (0.47-1.13)	0.91 (0.56-1.26)	0.29 (0.03-0.61)	1.88 (1.01-2.44)	0.07 (0.03-0.15)	0.13 (0.06-0.20)	2.24 (1.56-2.97)	0.45 (0.24-0.71)	0.54 (0.38-0.71)	0.86 (0.48-1.27)	0.14 (0.08-0.20)	0.32 (0.19-0.43)	0.12 (0.02-0.28)	0.66 (0.06-0.94)	1.37 (0.08-3.77)	8.29 (6.59-10.56)
Rio Grande do Sul	0.67 (0.41-1.00)	0.97 (0.60-1.32)	0.11 (0.02-0.24)	1.79 (1.03-2.32)	0.03 (0.02-0.05)	0.26 (0.13-0.39)	2.26 (1.62-2.92)	0.40 (0.21-0.63)	0.49 (0.31-0.65)	0.74 (0.41-1.12)	0.15 (0.08-0.27)	0.28 (0.18-0.36)	0.06 (0.02-0.14)	0.62 (0.05-0.86)	1.48 (0.07-4.06)	8.13 (6.50-10.31)

(Table 6 continues on next page)

(Continued from previous page)

Rondônia	0.49<br>(0.29-0.77)	0.87<br>(0.52-1.23)	0.17<br>(0.02-0.38)	1.69<br>(0.92-2.23)	0.04<br>(0.02-0.08)	0.12<br>(0.06-0.19)	2.32<br>(1.69-3.00)	0.41<br>(0.22-0.66)	0.50<br>(0.33-0.68)	0.67<br>(0.35-1.02)	0.10<br>(0.06-0.16)	0.27<br>(0.18-0.34)	0.08<br>(0.02-0.20)	0.61<br>(0.05-0.87)	1.42<br>(0.07-3.91)	7.70<br>(6.05-9.99)
Roraima	0.50<br>(0.3-0.76)	0.60<br>(0.35-0.86)	0.14<br>(0.02-0.31)	1.32<br>(0.75-1.71)	0.04<br>(0.02-0.08)	0.10<br>(0.05-0.15)	1.94<br>(1.42-2.48)	0.39<br>(0.22-0.58)	0.46<br>(0.31-0.60)	0.58<br>(0.31-0.87)	0.09<br>(0.05-0.13)	0.20<br>(0.13-0.26)	0.06<br>(0.01-0.15)	0.43<br>(0.04-0.61)	1.16<br>(0.06-3.13)	6.33<br>(5.03-8.15)
Santa Catarina	0.58<br>(0.34-0.87)	0.79<br>(0.49-1.13)	0.10<br>(0.02-0.24)	1.64<br>(0.94-2.15)	0.03<br>(0.02-0.05)	0.20<br>(0.10-0.30)	2.21<br>(1.62-2.86)	0.37<br>(0.20-0.59)	0.48<br>(0.30-0.66)	0.47<br>(0.24-0.75)	0.13<br>(0.07-0.22)	0.26<br>(0.17-0.34)	0.06<br>(0.02-0.14)	0.59<br>(0.05-0.85)	1.39<br>(0.07-3.88)	7.42<br>(5.86-9.57)
São Paulo	0.62<br>(0.37-0.92)	0.93<br>(0.56-1.29)	0.07<br>(0.02-0.16)	1.81<br>(0.99-2.37)	0.03<br>(0.02-0.04)	0.23<br>(0.11-0.34)	2.8<br>(2.04-3.59)	0.41<br>(0.22-0.69)	0.48<br>(0.27-0.67)	0.61<br>(0.3-0.95)	0.16<br>(0.09-0.26)	0.29<br>(0.18-0.38)	0.06<br>(0.02-0.12)	0.67<br>(0.06-0.95)	1.57<br>(0.08-4.18)	8.38<br>(6.69-10.57)
Sergipe	0.66<br>(0.40-1.00)	0.82<br>(0.48-1.17)	0.21<br>(0.03-0.45)	1.71<br>(0.98-2.23)	0.06<br>(0.03-0.12)	0.15<br>(0.08-0.22)	2.50<br>(1.87-3.16)	0.45<br>(0.24-0.69)	0.52<br>(0.36-0.68)	0.82<br>(0.46-1.23)	0.12<br>(0.08-0.19)	0.28<br>(0.17-0.36)	0.10<br>(0.02-0.22)	0.57<br>(0.05-0.8)	1.36<br>(0.07-3.71)	8.10<br>(6.53-10.08)
Tocantins	0.68<br>(0.41-1.03)	0.93<br>(0.55-1.30)	0.24<br>(0.03-0.51)	1.77<br>(0.97-2.35)	0.05<br>(0.03-0.12)	0.11<br>(0.05-0.17)	2.70<br>(2.00-3.44)	0.40<br>(0.22-0.64)	0.49<br>(0.33-0.66)	0.83<br>(0.46-1.25)	0.11<br>(0.07-0.16)	0.29<br>(0.19-0.38)	0.10<br>(0.02-0.23)	0.63<br>(0.05-0.91)	1.49<br>(0.08-4.12)	8.43<br>(6.64-10.81)

Data in parenthesis are 95% Uncertain Intervals (95%UI).
